# How allosteric mutations control ligand binding in Lipocalin protein: odorant binding protein as a test case

**DOI:** 10.1007/s00018-025-05777-8

**Published:** 2025-06-23

**Authors:** Maxence Lalis, Lucie Moitrier, Miriam Jäger, Cornelia Meinert, Marine Brulé, Christine Belloir, Nykola C. Jones, Søren V. Hoffmann, Sébastien Fiorucci, Steffen Wolf, Loïc Briand, Jérémie Topin

**Affiliations:** 1 Institut de Chimie de Nice UMR7272, Université Côte d’Azur, CNRS, 28 Avenue Valrose, Nice, 06108 France; 2 Centre des Sciences du Goût et de l’Alimentation, Institut Agro, CNRS, INRAE, Université de Bourgogne, Dijon, F-21000 France; 3 Biomolecular Dynamics, Institute of Physics, University of Freiburg, 79111 Freiburg, Germany; 4 Department of Physics and Astronomy, Aarhus University, Aarhus, 8000 Denmark

**Keywords:** Molecular dynamics, Site-directed mutagenesis, Circular dichroism, Isothermal titration calorimetry, Lipocalin, Odorant binding protein

## Abstract

Lipocalins are a family of proteins found in mammals that are essential for the binding and transport of various molecules, but the mechanisms underlying their target recognition are still unclear. To answer this question, we studied odorant-binding proteins (OBPs), a specific type of lipocalin involved in chemical communication and olfaction. Using an integrative approach combining numerical modelling and experimental validation, we identified key structural regions that regulate the entry of molecules into the binding pocket. Modification of these regions disrupts molecular recognition, highlighting their importance for function. In addition, we found that changes in distant parts of the protein influence binding, shedding light on allosteric mechanisms. These results advance our understanding of lipocalin function and open up avenues for the design of proteins with targeted binding properties.

**Supplementary Information:**

The online version contains supplementary material available at 10.1007/s00018-025-05777-8.

## Introduction

Lipocalins form a group of small, soluble proteins that play a crucial role in the transport, storage or sequestration of small molecules in mammals [1], that recognize a wide range of ligand [2–4]. Their ability to bind a wide range of lipophilic and other organic compounds underlines their essential role in mammals [5, 6]. They share a conserved tertiary structure, known as the lipocalin fold, which consists of an eight-stranded antiparallel β-barrel enclosing an internal binding cavity designed as the calyx [7–9]. However, the translocation process involved in ligand binding in lipocalin is still poorly understood [10], and this lack of knowledge limits the full use of lipocalins for potential applications. Advances in research into the control of the translocation mechanism could open up new avenues for the use of lipocalin in various biotechnology applications such as diagnostics, quality control and therapeutics [11–15].

**Fig. 1:**
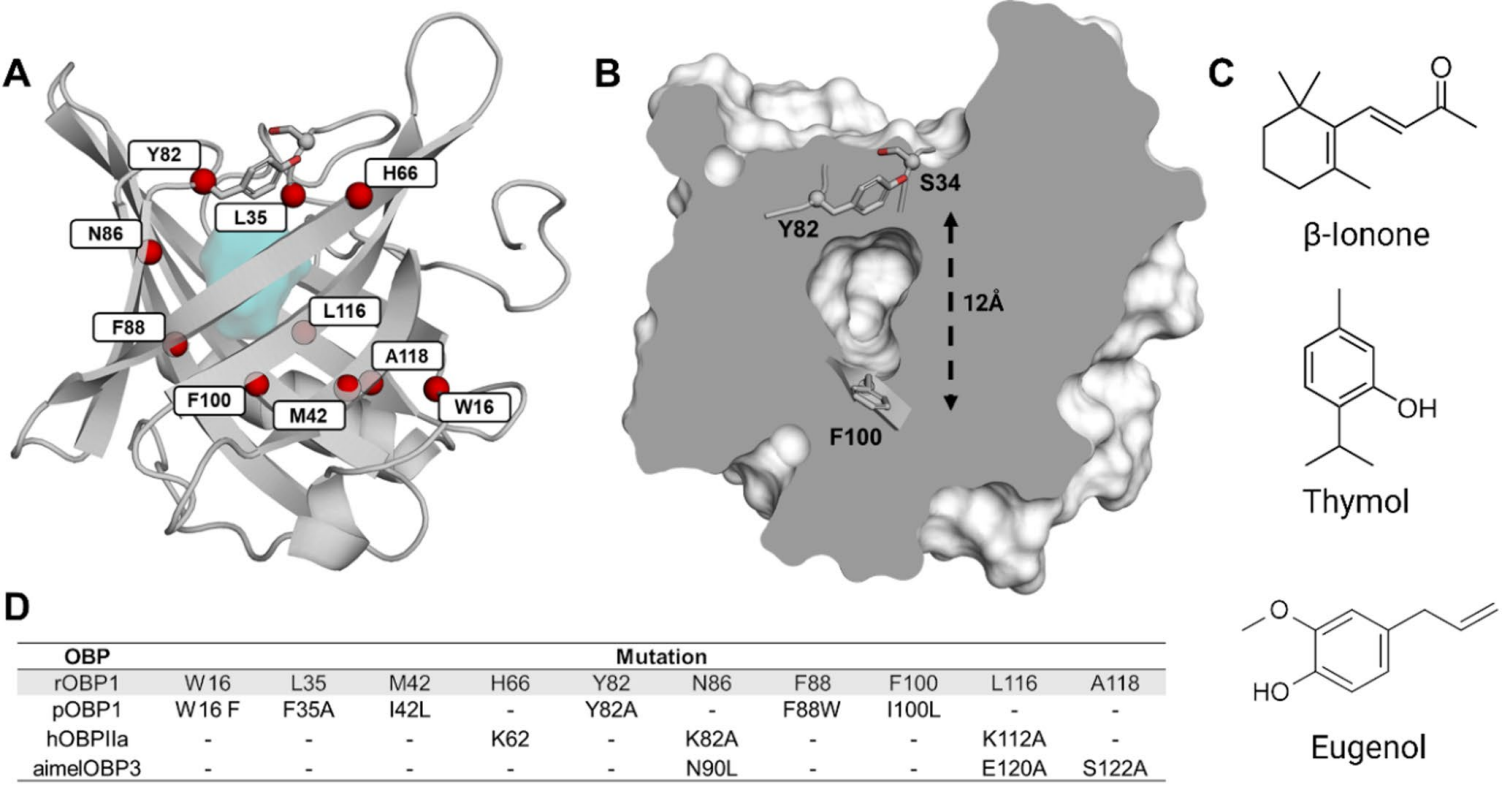
Structure of rOBP1 and position of the known mutants. (**A**) Cartoon representation of rOBP1 (pdb code 3fiq [27]). Positions previously subjected to point mutation are denoted by red spheres. The hydrophobic calyx appears as a light blue surface in the core of the protein. The two amino acids (Y82 and S34) predicted to act as a gate controlling ligand-binding are represented as sticks with carbon and oxygen atoms in grey and red, respectively. (**B**) Sliced surface representation of rOBP1 in the same orientation as A. The hydrophobic calyx appears as a void in the center of the protein. (**C**) Lewis representation of the molecules studied in this work. (**D**) Table summarizing positions which have been subjected to mutation in different mammalian OBPs

To investigate the ligand translocation pathway, we used mammalian odorant binding proteins (OBPs) as a model for the lipocalin family [16]. Elucidation of OBP structures has shown that this protein family adopts a similar fold with a large hydrophobic cavity in its core (Fig. 1A) [17, 18]. These proteins are involved in various functions, including removing odorants from the olfactory epithelium [19], implicated in the immune response [20], or scavenging free radicals in the nasal mucosa [21].

For OBPs, the calyx has been shown to be involved in the capture and transport of odorants as the presence of this hydrophobic cavity is a key feature that allows reversible binding of odorants [22, 23]. Previous studies combining site-directed mutagenesis with in vitro assays have demonstrated that the binding specificity of OBPs could be largely affected by replacing amino acids in the calyx (Fig. 1A, D and table S1). In addition, post-translational modifications at residues not directly involved in ligand recognition could also affect the different binding affinities of OBPs for specific ligands [24, 25], suggesting an allosteric modulation of the binding. However, the binding pathway of odorants to OBPs calyx remains relatively under explored, particularly regarding the molecular mechanisms that govern the entry and exit of ligands from the binding pocket. Structural studies of OBP revealed that the cavity inside the β-barrel structure is not directly exposed to the surrounding solvent [17, 22] (Fig. 1 and fig. S1). This implies the existence of a dynamic process inducing a protein reorganisation to create a channel through which odorants will diffuse. However, this translocation pathway has never been observed in structural studies in which the calyx remains occluded from the solvent (fig. S1). Molecular dynamics simulations have suggested that a conserved tyrosine (Y82 in Fig. 1) controlled the access to the calyx [26]. Despite its high conservation (82%) in OBPs and other lipocalins, the precise molecular mechanisms governing the binding pathway, and its role in different lipocalins remain to be fully elucidated. Moreover, as most site-directed mutagenesis has been performed inside the calyx and only a few in the vicinity of the gate (Fig. 1A, D), possible allosteric effects on ligand binding remain unknown.

In this study, we performed extensive equilibrium MD simulations [28–30] to decipher the mechanism of odorant binding to OBPs. OBPs from rat (rOBP1) and pig OBP1 (pOBP1) were considered for in silico experiments. This step provides molecular insights into the odorant binding pathway for 3 known rOBP1 binders [31] (Fig. 1C) and one known binder (2,6-dimethyl-7-octen-2-ol) for pOBP1 [32]. Additionally, we performed dissipation-corrected targeted MD simulations (dcTMD) [33, 34] together with pathway clustering [35, 36] to determine the free energy profile underlying the binding and unbinding process, as well as friction factors that reveal ligand hydration shell changes. The simulations revealed a conserved pathway linking the bulk to the calyx.

In order to confirm that odorants follow this single pathway to reach the binding site, a double mutant was constructed to block the gate in closed conformation. Residues serine34 and tyrosine82 were mutated to cysteine to create a disulfide bridge that would close the OBP gateway and prevent binding to odorants. The fold and stability of this mutant was assessed by synchrotron radiation circular dichroism spectroscopy [37] and confirmed the formation of a disulfide bridge. The loss of function of this mutant was assessed by isothermal calorimetry (ITC) experiments [38]. Finally, guided by protein network analysis, we identified an allosteric site regulating odorant binding, which was confirmed through mutagenesis and ITC measurements. Altogether, our results provide a detailed description at the atomic level of the binding process in OBP and show how specific mutations could alter the binding process by blocking the translocation pathway or disturbing protein dynamics. By analyzing the conservation of these residues we propose that this binding mechanism is a conserved feature across the lipocalin family.

## Materials and methods

### Molecular Dynamics

All unbiased simulations were carried out using the AMBER20 package and the FF14SB force field [39]. Protein structures of rOBP1 and pOBP1 were taken from X-ray structures (accession code: 3fiq [27] and 1e00 [22]), the protonation state at pH 7.5 of their amino acids was computed using the H + + webserver [40]. Ligands were optimized at the B3LYP/6-31G* level using Gaussian 19 [41]. Partial charges for each atom were then obtained using the antechamber module. Finally, ligand parameters were obtained from the Generalized Amber Force Field (GAFF2).

In order to increase the sampling of binding events, 5 ligands were introduced randomly around the protein (fig. S2). The simulation box was completed using TIP3P water molecules and neutralized using Na^+^. The total system is made up of 35,779 atoms, in a 358,566 Å^3^ periodic box. Shake algorithm was used to apply bonds involving hydrogen atoms allowing to set a time step of 2 fs during MDs simulations.

Systems were minimized by 10,000 minimization steps and 20 ps of MD simulations at 100 K using a restraint of 20 kcal∙mol∙Å^−^² on the protein were performed, followed by four rounds of 10,000 minimization steps reducing the restraints by 5 kcal∙mol∙Å^−^² at each round, with 20 ps MD simulation in between each step. Then, the systems were slowly heated to 300 K over a period of 20 ps. Equilibration runs were continued over 20 ns prior to the production simulations. Production simulations of 200 ns were extended if no binding event was observed. The entrance of the ligand inside the binding cavity was monitored by its distance to the F100 residue for rOBP1 (fig. S3). During the 10 replicas, rOBP1 underwent small fluctuations (RMSD_backbone_ < 2 Å) showing great stability (fig. S4 and S5) of the OBP fold. Similar behavior was observed for pOBP1-wt (fig. S6). During the MD simulations, the C-terminal part is stabilized near the calyx by a disulfide bond between two cysteine: residues 79 and 170 in rOBP1 and 63 and 155 in pOBP1.

Clustering was done using the DBSCAN clustering algorithm [42] with a minimum number of 50 points required to form a cluster and an epsilon of 5, which is the distance cutoff between points required to form a cluster. A minimum of 50 points corresponds to a cluster that persists for 10 ns during simulation. Mass-weighted RMSD was used to define distance between frames. RMSD distance cut-off of about 5 Å was chosen to explore the same binding pose while allowing moderate internal flexibility and various orientations.

### dcTMD simulations and MoSAIC-based trajectory clustering

During dissipation corrected target MD (dcTMD), a pulling force is applied along a reaction coordinate (x) using a displacement distance constraint. This method yields both the free energy profile ΔG(x) and the friction field Γ(x). The friction captures dynamical interactions with degrees of freedom that are not directly involved in the free energy landscape and allows monitoring the hydration of the binding site [34].

Dissipation-corrected targeted MD simulations were carried out using the PULL code in Gromacs v2020.3 [43] with the Amber14 forcefield and the TIP3P water model. Ligand parameters were imported from AMBER using acpype [44]. As a starting structure, one of the final simulation structures with bound β-ionone was used, which was first equilibrated with a steepest descent minimization, followed by a 100 ps simulation in the NPT ensemble at 300 K and 1 bar, each with positional restraints of 2 kcal∙mol∙Å^−^² on protein and ligand heavy atoms. The full system was then subjected to another round of steepest descent minimization, followed by another 100 ps in the NPT ensemble with protein and ligand positional restraints, finally followed by 10 ns of fully unbiased equilibration.

From the final simulation structure, 200 statistically independent replicas were generated by the independent attribution of atomic velocities. After 10 ps of heating to 300 K in the NPT ensemble with positional restraints on protein and ligand heavy atoms, each simulation replica was subjected to another 10 ps of short equilibration without positional restrains, but with a constant distance constraint between the centers of masses of the β-ito another 10 ps of short equilibration without positional restrains, but with a constant distance constraint between the centers of masses of the β-ionone heavy atoms and 26 C_α_-atoms of the protein’s central β-sheet. Finally, all 200 simulation replicates were continued for 2.5 ns, replacing the constant distance to a constant velocity constraint with a pulling velocity of 1 m∙s^−1^.

The resulting trajectories were clustered based on a principal component analysis of protein-ligand contacts [36, 45] using the first four principal components to calculate trajectory-pairwise similarities s_ij_. Unbinding paths were revealed via Leiden community detection [46] using the MoSAIC tool [47], with the constant Potts model as objective function. The constant Potts model requires only the definition of a single parameter, the resolution parameter γ, thereby requiring minimal user input (see the supplementary materials for details). Here, we use a resolution parameter $\:\gamma\:=median\left({s}_{ij}\right)$, which has to be shown to be effective for path separation. Work profiles of trajectories of the largest cluster were calculated using dcTMD [33, 36], yielding free energies and friction factors along the unbinding path.

### Network and MoSAIC Analysis of protein dynamics

To assess the global communication profile of our protein, we analyzed the global metapath using webPSN webserver with default settings [48], which represents the shortest communication pathway between all pairs of residues in the network. The global metapath offers a comprehensive view of the overall communication landscape and helps to pinpoint critical residues that contribute to the protein’s function and regulation.

We also evaluated the correlated dynamics of the OBP from MD simulations with MoSAIC [47]. As input for the analysis we determined pairwise minimal residue contact distances with a heavy atom distance cut-off of 4.5 Å [45]. Then, we calculated the Pearson correlation ρ between the distances and clustered the resulting pair-wise correlation matrix [47] (see supplementary material for more details). We identified well-connected clusters using a resolution factor γ=0.2. This threshold was chosen to ensure that the resulting clusters represent functionally meaningful groups of residues. Lower thresholds resulted in clusters with weak internal correlation, which restricted their interpretability in terms of collective motion.

### Sequence alignment

Sequences were taken from Pelosi et al. [16]. Alignment was made with Jalview using the Mafft algorithm and used to calculate sequence conservation with Jalview [49] (Supplementary files 1). Weblogo were constructed using Weblogo3 [50] and phylogenetic tree with the online webserver iTOL [51].

### rOBP1 production and purification

Recombinant rOBP1-wt (wild-type) and mutants were produced as previously described [52], see fig. S7 for more details about the sequence and construction. Briefly, the cDNA sequence encoding the mature rOBP1 (Gene name: Obp1f, UniProt ID: P08937) was synthesized with codon usage optimized for expression in *E. coli* (Genewiz, Leipzig, Germany) and cloned into the *BamH*I and *Hind*III restriction sites of the pQE31 plasmid (Qiagen, Hilden, Germany). The resulting expression plasmid pQE31-rOBP1 encodes a fusion protein composed of an N-terminal His6-tag, followed by rOBP1 (His16-Gln172). Mutants were prepared using customized site-directed mutagenesis by Genewiz (Leipzig, Germany). The plasmids were subsequently introduced into *E. coli* M15 [pREP4] cells (pQE expression system, Qiagen). The expression of rOBP1-wt and mutants were induced with 0.2 mM isopropyl β-D-1-thiogalactopyranoside (IPTG) for 3 h at 37 °C in Terrific Broth medium supplemented with antibiotics.

Recombinant His-tagged rOBP1-wt and mutant proteins were purified as previously described using standard Nickel-affinity chromatography. To remove potentially bound contaminants, the purified rOBP1-wt and mutant proteins were extensively dialyzed for 24 h against a buffer (100 mM sodium phosphate, pH 7.5) containing 5% (v/v) acetonitrile at 4 °C. After an additional 24 h of dialysis against the same buffer in the absence of acetonitrile, fractions containing rOBP1 proteins were analyzed by SDS‒PAGE, pooled and stored at −20 °C. All buffers used for purification were prepared with HLPC-grade water. The purity of purified proteins was assessed by SDS‒PAGE, and their concentrations were determined with UV spectroscopy.

### Recombinant rOBP1 characterization

The purity of rOBP1-wt and the mutants was assessed by SDS‒PAGE, and concentrations determined by spectrophotometry, measuring the absorbance at 280 nm and using an extinction coefficient of ε_280_ = 16,305 M⁻^1^∙cm⁻^1^. The oligomeric state of rOBP1 was analyzed by size exclusion chromatography using a 24-mL bed volume Superdex 75 10/300 GL column (GE Healthcare). The column was equilibrated in a solution containing 100 mM sodium phosphate and 150 mM NaCl at pH 7.5 at 1 mL∙min⁻^1^. Bovine serum albumin (67 kDa), chicken egg ovalbumin (43 kDa), bovine ribonuclease A (13.7 kDa), and aprotinine (6.5 kDa) purchased from Sigma, were employed as standards (fig. S8).

### Isothermal titration calorimetry

Isothermal titration microcalorimetry (ITC) experiments were carried out at 25 °C using a VP-ITC microcalorimeter (Malvern Instruments, Malvern, UK) as previously described [52, 53]. Protein solutions (25 µM in 50 mM sodium phosphate at pH 7.5) were thoroughly degassed before the experiments. Odorant solutions (250 µM in 50 mM phosphate buffer, pH 7.5) were injected into 25 successive 10 µL aliquots. Binding parameters were calculated by fitting the values to a single site binding model through nonlinear regression using Microcal Origin^®^ software.

### Synchrotron radiation circular Dichroism spectroscopy

Electronic Circular Dichroism (ECD) and absorbance spectra of rOBP1 were recorded at the AU–CD beam line on ASTRID2 at Aarhus University (Denmark) [54]. rOBP1-wt and rOBP1 mutant (S34C Y82C) samples were diluted in a 100 mM sodium phosphate solution at pH 7.5 to achieve a concentration of ~ 1 mg∙ml^−1^. All spectra were measured using a nominally 0.1 mm path length cell (quartz Suprasil cell, Hellma GmbH & Co., Germany), with the precise length determined using interferometry measurements [55]. The concentration of the samples were determined from the absorbance at 205 nm [56], which is measured simultaneously with the CD spectrum. Spectra were recorded at 25 °C in triplicate in the wavelength range of 170 to 280 nm in steps of 1 nm and a dwell time of 2 s, with a corresponding baseline triplicate scans measured of the buffer alone. Finally, the percentages of secondary structure were obtained by a deconvolution of the spectra using BeStSel [57] webserver.

Temperature scans were carried out in a Starna type 31B cell, with a nominal pathlength of 0.1 mm, with the full CD spectrum measured in triplicate at each set temperature, in steps of 2.5 °C and an equilibration time of 1 min before scanning. Scans of the reference baseline measured with the same protocol were subtracted for each temperature.

A summary of the different methodologies can be found as fig. S9.

## Results

### Ligand entry mechanism from MD simulations

To clearly identify ligand translocation in OBP, we performed MD simulations to track the entry of three ligands, β-ionone, eugenol and thymol (Fig. 1C), into the calyx of rOBP1-wt. A total of 15 simulations, resulting in an aggregated simulation time of 22 µs, were done. We considered the ligand in the cavity when the distance between its center of mass and the center of mass of residue F100, forming the cradle of the cavity, was less than 5 Å. During these simulations, 10 binding events were observed (fig. S3). The same protocol was applied to pOBP1 and 5 binding events recovered through 10 independent simulations.

**Fig. 2:**
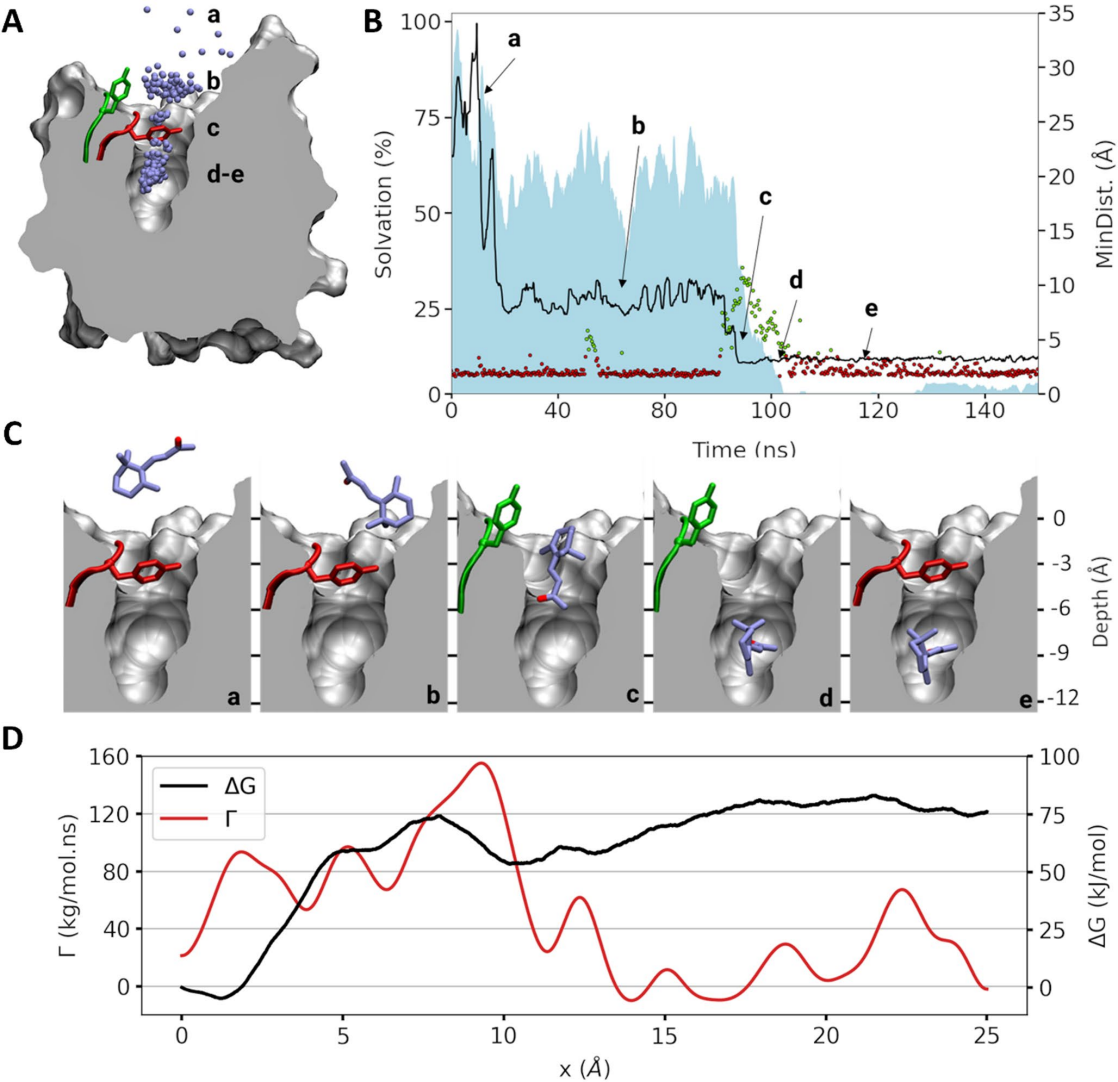
Translocation mechanism of β-ionone in rOBP1. (**A**) Sliced surface representation showing the center of mass of β-ionone at every timestep in lavender balls. Y82 is shown either in red or green for closed or open state respectively. (**B**) Evolution of the distance between β-ionone centers of mass and the center of mass of the F100 lateral chain drawn as a black curve separated in different areas. Zone (a) represents the binder in water to the contact of rOBP1. Zone (b) represents the partial stabilization in the pocket, (c) the entry, (d) β-ionone in the cavity with the gate open and (e) in the cavity with the gate closed. The blue area shows the percentage of ligand solvation during the binding process normalized to the solvation of the ligand in water. The distance between the hydroxyl group of Y82 and carbonyl of the backbone of S34 is represented in dots. Red dots correspond to the closed state (< 3.4 Å) and green to the open state. (**C**) Close view of the binding process with representative position for each zone defined in B. Carbons atoms of β-ionone are colored in lavender and oxygen in red. Y82, and its loop are colored according to its opening (red closed, green open). (**D**) Free energy (red) and friction (black) estimate from dcTMD unbinding simulations. The free energy curve exhibits a local minimum at x = 10 Å, corresponding to state c in the unbiased simulations shown above. The peak in friction at x = 9 Å coincides with the decrease in ligand hydration

The MD simulations revealed a conserved pathway for all the considered ligands. The analysis of a prototypical binding trajectory is presented in Fig. 2. To access the binding cavity, ligands diffuse through a highly dynamic gate formed by the conserved Y82 and the S34 (Fig. 2A). The aromatic tyrosine acts as a hydrophobic cap, occluding the cavity from the solvent. On the basis of the side chain fluctuation and odorant solvation, the binding pathway is divided into five subsequent steps: contact-closed, pocket-closed, entry-open, binding site-open, and binding site-closed (Fig. 2A-C, a, b, c, d, e, respectively).

The first step (contact-closed, a) involves rapid contact with the vicinity of the gate and partial dehydration of the ligand (~ 25%) and no specific interaction with the protein is clearly observed.

The second step (pocket-closed, b) consists of stabilisation of the ligand in the vicinity of the gate for ~ 70 ns and stable solvation at 60%. Ligand migration seems to depend on the conformation of the Y82 side chain, whose closed form is stabilised by a hydrogen bond with the backbone of S34, which is located at the opposite side of the entrance. This interaction must be broken to allow the ligand to enter the protein. Simulations of the apo form of the protein revealed spontaneous opening and closing of the gate, even in the absence of ligand (fig. S10A). This observation suggests that the dynamic of the door opening is independent of the presence of a ligand in the vicinity of the gate and that the binding follows a conformational selection rather than an induced fit process.

The third step (entry-open, c) begins with the breaking of the hydrogen bond between S34 and Y82, increasing the degree of freedom of the loop and causing the switching of Y82, which opens the cavity and allows entry of the ligand. This migration is accompanied by a sharp drop in ligand solvation to less than 20%. Ligand entry into the calyx protein is rapid and appears to be irreversible on the time scale of the simulation as we do not observe any unbinding event.

When the ligand enters the cavity, we observe almost complete solute dehydration (step d in Fig. 2, binding site open). Then, the Y82 sidechain undergoes a rotation that induces the closing of the cavity (step e in Fig. 2A, B and C, binding site-closed). The ligand does not adopt a specific binding position, and appears to be mostly stabilized through hydrophobic contact (table S2) as observed in a recent study [58]. The simulations recover most of the ligand interactions with amino acids previously identified as playing a key role in binding (table S2). Finally, simulations realized on pOBP1 unveil a similar pathway for the dihydromyrcenol binding (fig. S11), suggesting a conserved binding pathway across mammalian OBPs.

Throughout the simulations, we observed a variable duration of the pocket-locked step, which depends on the gate opening. It appears to be a stochastic event, independent of the ligand presence (fig. S10C). Stabilization next to the gate does not necessarily lead to binding. If the gate does not switch to the open conformation (Fig. 2), the ligand cannot access the calyx of the protein and will dissolve back into the solvent (fig. S10C).

During the simulations, no unbinding event was observed (fig. S3). Thus, in order to sample the unbinding pathway, we performed dcTMD simulations, resulting in 200 unbinding events. Trajectory clustering (fig. S12) revealed that 130 of the 200 trajectories unbind along the same pathway as our unbiased binding path. The resulting free energy profile in Fig. 2D displays a local minimum at a distance of 10 Å from the binding site, which perfectly aligns with the pocket-locked position shown in Fig. 2C. In agreement with this observation, the peak in friction ${\Gamma\:}$ at a distance of 9 Å agrees excellently with the decrease in ligand hydration. The respective free energy difference between the bound state and this local minimum is on the order of ca. 55 kJ∙mol^−1^, i.e., 13.5 kcal∙mol^−1^, which corresponds roughly to a nanomolar affinity for β-ionone, which is in reasonable agreement with our experimental values. We note that the remaining 25 kJ∙mol^−1^ for complete unbinding into the solvent, agree well with β-ionone’s logP of 4.

The simulations thus lead to a complete description, at the atomic level, of the ligand binding pathway and guide site-directed mutagenesis experiments combined with ITC assays to assess the role of the gate and amino acids in its local environment.

## Site-directed mutagenesis and ITC experiments confirm the translocation pathway

### Confirmation of gating via mutagenesis

The simulations revealed the hydrogen bond between Y82 and S34 to be essential in controlling the ligand binding. We sought to strengthen the interaction between these two residues by creating a disulfide bond between these two positions which will lock the protein in the closed state and would prevent the ligand from binding to the calyx. This methodology has already been successful to abrogate ligand binding in lysozyme [59]. The distance between the two residue side-chains is fluctuating between 3 and 5 Å, such that it should be favorable to form a disulfide bond [60, 61]. We first generate a model of the double mutant Y82C-S34C with a disulfide bond in between the two cysteine residues (fig. S10B). No large change in the overall structure appeared during the MD simulations compared to the rOBP1-wt (fig. S4) suggesting that this double mutant should not affect rOBP1 folding.

**Fig. 3:**
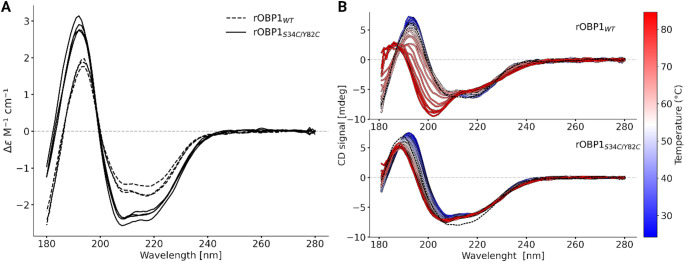
Analysis of the rOBP1 mutant folding and stability. (**A**) Circular dichroism spectra of rOBP1-wt and double mutant S34C and Y82C in dashed and plain lines respectively. Three and four independent measurements have been made for the wt and double mutant, respectively. (**B**) Temperature scan plots displayed as a function of temperature, with overlaid CD spectra recorded at temperatures ranging from 25 °C to 85 °C. The dotted black lines represent the spectra at 25 °C, while the series of colored lines correspond to spectra at each incremental temperature

We further confirmed these in silico results by investigating the folding of the double mutants by synchrotron radiation circular dichroism spectrometry [62, 63]. UV CD spectra recorded down to 180 nm for both wt and mutant are similar (Fig. 3A). The deconvolution analysis of the different spectra shows that the percentage of secondary structures are not significantly affected by the mutations (fig. S13) confirming that that these double mutations have a minor impact on the protein secondary structure. 

Finally, the stability of both the wt and the double mutant was assessed through measurements of CD spectra with increasing temperature [64]. For rOBP1_wt_ the protein undergoes a clear change with temperature, unfolding and becoming more unordered, with a transition temperature of 70 °C. The unfolding of the wt was reversible, with the original spectrum largely recovered when returning to 25 °C (dotted black line Fig. 3B). The double mutant showed a higher stability than the wt, with a smaller change in the spectrum at higher temperatures, though the folding was not completely recovered when back at 25 °C (Fig. 3B). Overall, these results suggest that the mutations do not affect the folding and that the two cysteines are forming a disulfide bridge that stabilizes the protein structure.

**Fig. 4:**
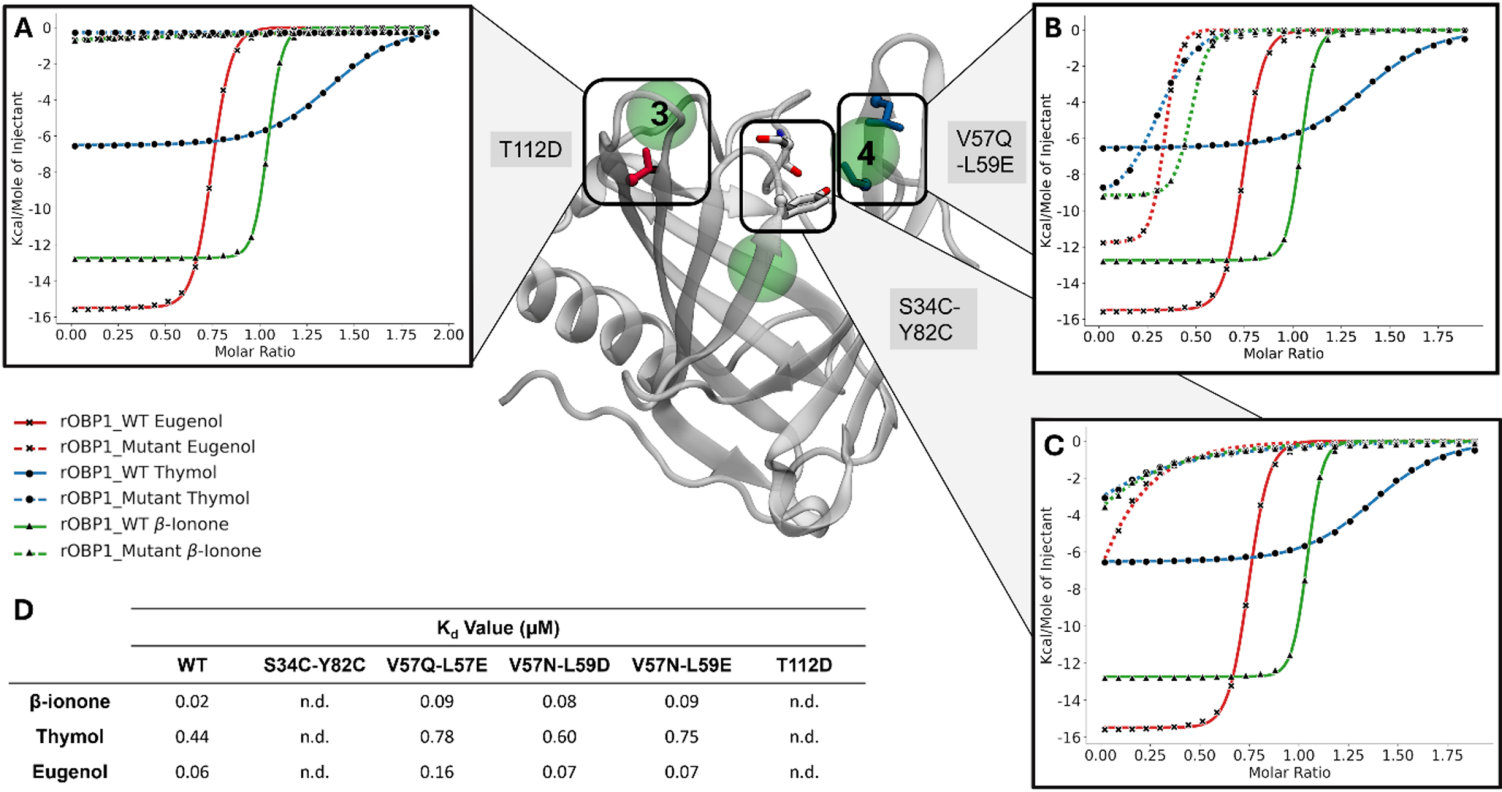
ITC assays on rOBP1 mutants in the local environment of the gate. Cartoon representation of rOBP1 with cluster position of the ligands near the gate shown as green spheres. Mutated amino acids are represented in different colors. All mutants were tested with the three ligands. (**A**) Left box, shows ITC assays results from mutation in the hydrophilic pocket 3 with T112 in red. (**B**) Right box shows experimental results on mutation in the identified hydrophobic pocket 4 with V57 and L59 in blue. For the sake of clarity, results for the mutant V57D/N and L59E/N are not shown. (**C**) Middle box, shows ITC assays results on the mutant at positions S34 and Y82, represented in grey. All experiments are realized on 3 binders of rOBP1: β-ionone, thymol, and eugenol. (**D**) Table summarizing the K_d_ values for all the experiments, n.d. means not determined

The response of the rOBP1 double mutant response to agonists stimulation was assessed by isothermal titration calorimetry (ITC). This double mutation resulted in a total loss of binding for the three odorants as no heat change was observed (Fig. 4), further emphasizing the crucial role of the two residues in controlling the odorant binding. Moreover, this loss of function demonstrates that this gate is the sole entry point to the calyx and also confirms that there is a unique path for the ligand to access the binding site of the mammalian OBP.

### Allosteric network analysis and distal mutation effects

We further investigated the local environment surrounding the gate. During the binding process, we regularly observe ligand transient stabilization at different positions around the protein. Ligands are stabilized in these pockets through weak interactions. Clustering analyses of the odorants’ positions revealed two clusters adjacent to the gate across all studied systems (Fig. 4, fig. S14). These two pockets exhibit significant differences in hydrophobicity. The first pocket “3”, contains a high proportion of conserved hydrophobic residues. In contrast, the second pocket “4”, is characterized by a high proportion of conserved hydrophilic residues. We propose that hydrophobic pocket ‘4’ is involved in partial dehydration and stabilization in step (b) (Fig. 2B). Simulations showed that L57 and V59 interacted significantly with odorants in this pocket.

Mutation of these residues to hydrophilic residues of variable size (V57Q/N and L59E/D) did not result in significant changes in binding affinities for none of the studied ligands (Fig. 4 and fig. S15). In all cases, the K_d_ remains within the same order of magnitude. However, we observed a compensation in the entropy-enthalpy balance: these quantities increased for β-ionone and eugenol, while they decreased for thymol (table S3). Thus, our results show that the modification of the hydrophobic properties of this pocket does not influence the binding affinity but induces a variation in the thermodynamic binding signature.

We next focused on the opposite hydrophilic pocket. The conserved residue T112 was mutated into aspartic acid to further increase the hydrophilicity [65]. No detectable heat change was measured, even at ligand concentrations in the millimolar range, indicating a complete loss of affinity for all ligands (Fig. 4 and Fig. S15). This suggests that T112 is essential for odorant binding.

**Fig. 5:**
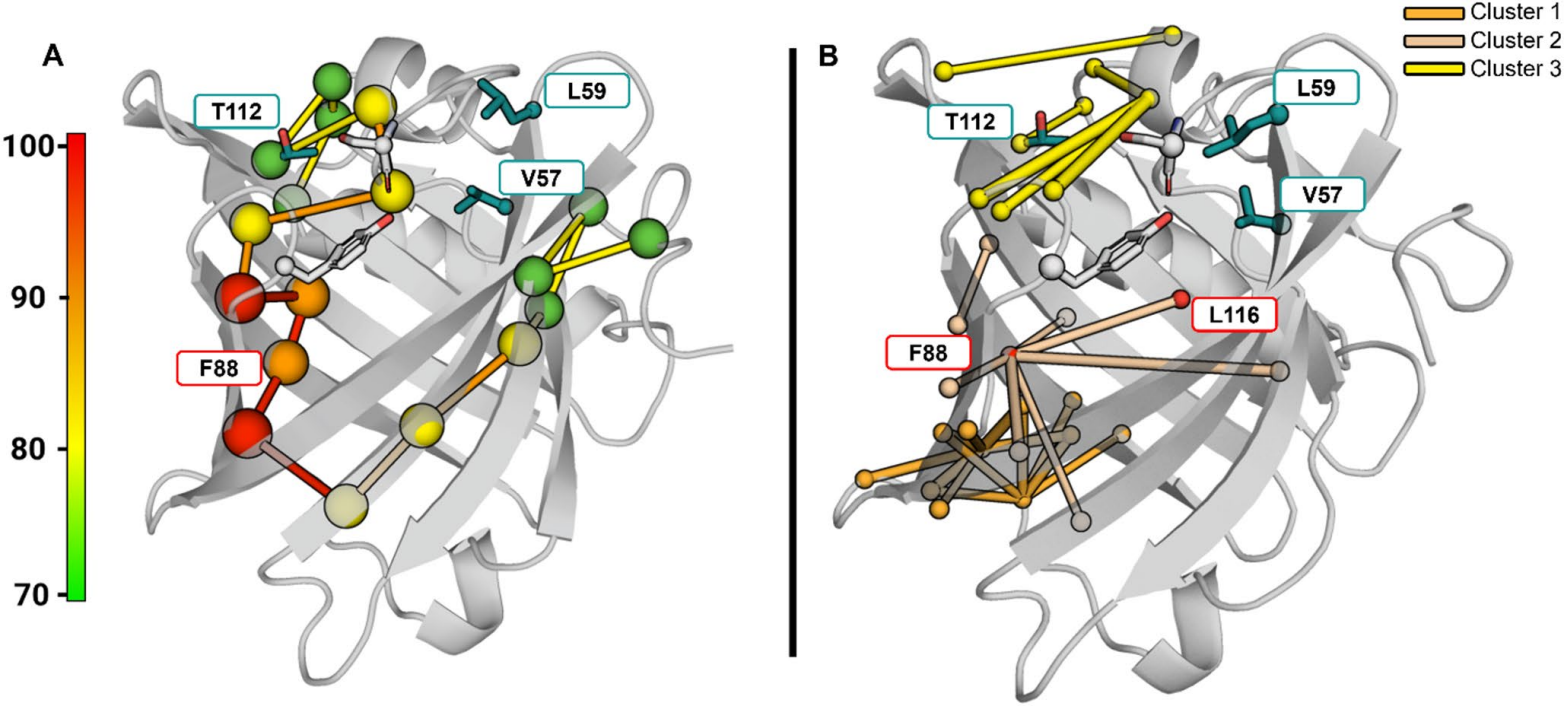
Structural communication signature in the OBP structure probed by network analysis. Mutated positions (T112, S34, Y82, L59, V57) are represented in licorice. Carbon atoms from the gate (S34 and Y82) are colored white, while V57, L59 and T122 are in cyan. Mutations made on residues boxed in red have been previously shown to abrogate the ligand binding. (**A**) Global metapath obtained on the crystallographic structure of rOBP1 (pdb code 3fiq [27]). Nodes and links are colored with a gradient from green (*I* = 70) to red (*I* = 100), where *I* = 100 is the most interacting node or link. Hubs are shown as spheres centered on the Cα-atoms and colored according to the average interaction strength of their links. Nodes and link with an interaction frequency *I* below 60 are not shown. (**B**) MoSAIC analysis results on all apo rOBP1-wt MD simulations. The highly correlated minimal distances appear as colored lines between the involved amino acid Cα-atoms according to the cluster classification. Interestingly, the two previously identified positions F88 and L116 belong to the same cluster (cluster 2 in fig. S16). On the other hand, V57 and L59 are not highly correlated to the rest of the protein, as seen from the analysis of crystallographic structure

As the previously identified pockets are distant from the binding site but still influence binding, we hypothesize that they are involved in allosteric communication with the OBP’s gate. To investigate this, we performed an interaction network analysis using PSNtools [48] on the crystallographic structure of rOBP1. This method allowed us to map allosteric communication between distant sites by identifying the shortest communication pathways through the interaction network (Fig. 5A). Residue T112 was found to be part of a communication network involving loops constituting the gate, and the adjacent α-helix. Disruption of this network may impair the dynamics of the gate. On the other hand, residues L59 and V57, which had minimal effect on binding, were not part of this pathway, highlighting their negligible role in odorant binding to rOBP1.

Additionally, we analyzed correlated motions of the protein during the MD simulations using MoSAIC. This analysis identified small communities of correlated movements, with rOBP1 showing limited large-scale correlations (fig. S16), consistent with previous findings [66, 67]. Notably, the third-largest community includes the gate, the adjacent α-helix, and residue T112, suggesting coordinated movements between the gate and the region surrounding this residue (Fig. 5B). This suggests that T112 would be involved in the allosteric regulation of gate dynamics, influencing the overall binding process. In contrast, residues V57 and L59 were not part of any major motion networks, further confirming their minimal contribution to the protein’s dynamics and binding mechanics.

Residue controlling the gating is highly conserved across lipocalin.

**Fig. 6:**
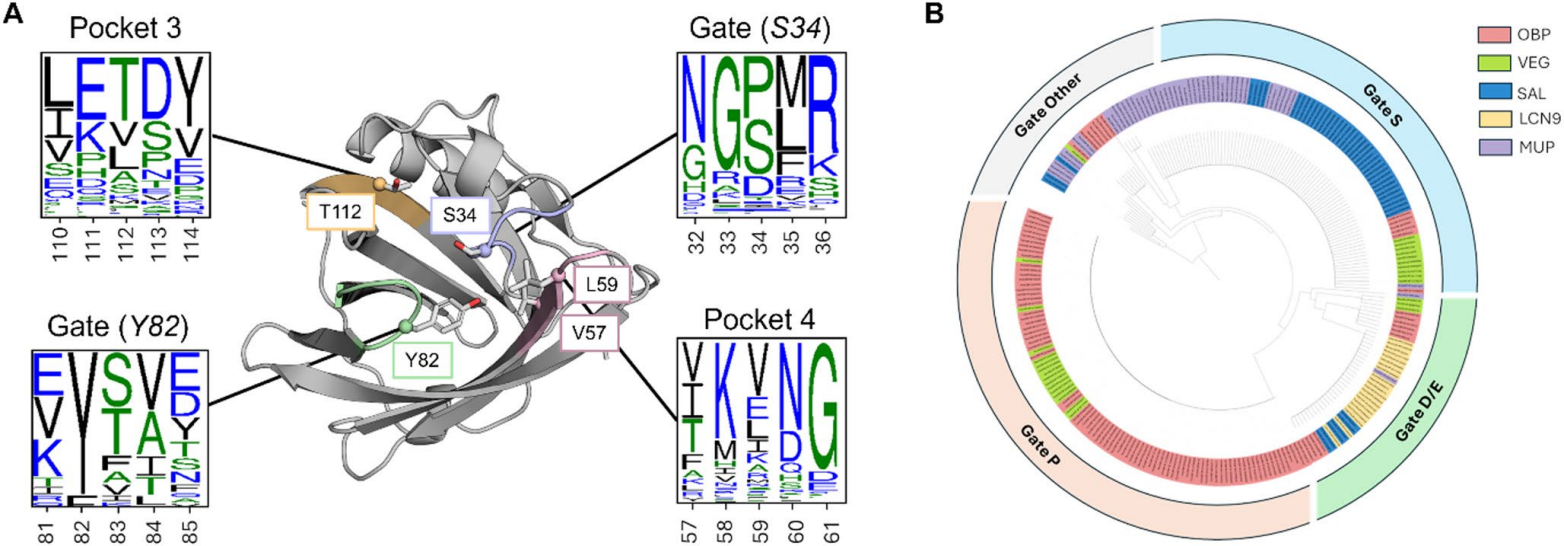
Lipocalin conservation analysis. (**A**) Residue conservation among 248 lipocalin proteins including 105 OBPs, 29 VEGs, 46 SAL, 21 LCN9 and 47 MUPs. In each frame, the consensus logo is colored according to the side-chain chemistry (blue black and green for charged, hydrophobic and hydrophilic respectively). The height of symbols indicates the relative frequency of each amino or nucleic acid at that position. (**B**) Hierarchical cluster tree based on the alignment of gate position (S34 in the case of rOBP1). Gates P, S, D/E indicate a proline, a serine, or either an aspartate or glutamate respectively. All other amino-acid types have been grouped under Gate Other

In this study, we observe that the binding and unbinding processes rely on the opening of a ‘gate’ formed by residues Y82 and S34. The gate remains closed thanks to a hydrogen bond between the hydroxyl group of the tyrosine and the carbonyl of the serine backbone. Once this hydrogen bond has been broken, the two opposing loops gain mobility and separate, creating an opening towards the calyx which allows the ligand to enter. This tyrosine residue is highly conserved (82%, Supplementary files 1) among OBPs and most lipocalins, suggesting that this mechanism may be a common feature of lipocalin folding (Fig. 6).

Tyrosine, with its hydroxyl group capable of acting as both a hydrogen bond donor and acceptor, plays a crucial role in controlling binding. Additionally, its aromatic ring, through hydrophobic interactions, helps occlude the calyx from the surrounding solvent, preventing hydration of the cavity. This feature facilitates the dehydration observed during ligand binding and may explain why tyrosine is highly conserved. Phenylalanine is an alternative to tyrosine in the lipocalin fold, further emphasizing the importance of a bulking residue occluding solvent entry [66]. Interestingly, substituting it with a smaller amino acid, such as alanine, abrogates binding [68]. Previous studies have demonstrated that ligand binding is primarily driven by enthalpic contributions, while the unfavorable entropic loss due to ligand restriction may be offset by the release of highly ordered water molecules within the binding cavity or an increase in the dynamics of protein side chains [69–71]. Substituting tyrosine with alanine, however, disrupts this balance, and might flood the cavity eliminating the favorable entropic gain associated with the release of water molecules [68]. In contrast, replacing the gate with a disulfide bridge enhances steric discrimination by permanently blocking access to the calyx, effectively hindering ligand recognition.

Interestingly, the opposite residue of the Y82, serine in the case of rOBP1, shows variation among the lipocalin family (Fig. 6A). Serine is predominant in most von Ebner’s gland proteins (VEGs) or salivary lipocalins (SALs) which are members of lipocalins involved in chemical communication [16], while proline is common to most OBPs (Fig. 6B). However, given that only the backbone is involved in this hydrogen-bonding gate, we would expect greater variability at this position. We hypothesize that this residue could have an impact on the dynamics of the gate, with a serine forming an additional hydrogen bond with its side chain, thereby reinforcing a more constrained conformation, and conversely, a proline favoring a more flexible conformation allowing for a broader recognition.

## Discussion

While previous studies have shown the role of the gate in protein binding, here we successfully locked the OBP into a closed state. Our findings demonstrate that inserting a disulfide bridge is an effective strategy for engineering non-responsive OBPs by permanently blocking access to the calyx, hindering ligand recognition. This strategy could be applied to any lipocalin fold. Furthermore, we reveal that the gate is the sole entrance to the binding pocket, emphasizing the importance of positioning the OBP with the gate facing the bulk solution in biosensor applications.

Through MD simulations, we also identified two sites of transient stabilization for the binders near the gate region, each exhibiting different polarities (fig. S14). One of these sites, pocket 3, contains a high proportion of conserved hydrophilic amino acids (Fig. 6A), which is involved in a network that includes the gate (Fig. 5). Notably, mutation of a key residue—T112 which is poorly conserved (Fig. 6A) —to aspartic acid abolishes ligand recognition by rOBP1, highlighting the role of this distal residue in the allosteric regulation of binding. This suggests that alterations to the hydrogen bond network may influence the binding of odorants to rOBP1. Network analysis has proven effective in identifying allosteric sites on protein [72] and highlights T112 as a critical node. Our results recover the observation that mutations at such identified distal sites can affect binding affinity, emphasizing the importance of long-range interactions in protein function [73]. This finding is further supported by Stepanenko et al., [74] who proposed the existence of an ionic network crucial for OBP dynamics. Their work underscores the importance of electrostatic interactions in maintaining the structural flexibility necessary for ligand binding. Moreover, residue T112 might be subject to phosphorylation [24]—a post-translational modification thought to regulate the binding specificity of OBPs. Phosphorylation at this site could provide a mechanism for the regulation of ligand binding.

Conversely, we identified a hydrophobic pocket (pocket 4 in Fig. 6A) where mutations do not directly influence the ligand affinity but modify the thermodynamic signature of binding (Fig. 4B, table S3). The conservation of this region and its high hydrophobic content exposed to the solvent suggest it may play a role in protein-protein interactions. Hydrophobic surface patches are well-known facilitators of such interactions [75], and OBPs are proposed to interact with olfactory receptors [76] or the endocytic receptor megalin [77]. This hydrophobic patch could thus be critical for mediating these interactions.

This patch might facilitate progressive change in the solvation shell of the ligand, affecting the thermodynamic signature [78]. This is further supported by the modeling of unbinding events (Fig. 2D). We observed that the greatest friction between the ligand and the protein coincided with its dehydration, emphasizing the importance of ligand solvation in the binding/unbinding process. These experiments provide a description of the energy along the ligand’s entry trajectory, making it possible to quantify the different stages of this migration.

## Conclusion

Protein binding pocket dynamics are key to determining interaction specificity and binding processes. Protein flexibility enables adaptation to different ligands and highlights the importance of considering internal motion in predicting binding properties and designing new compounds. Binding pockets are defined by surrounding amino acid residues, and flexibility allows pockets to open, close, or adjust to accommodate ligands. Protein pocket dynamics were categorized in 5 different classes: the formation or disappearance of (1) subpockets and (2) adjacent pockets, (3) pocket breathing due to molecular fluctuations, (4) the opening or closing of channels or gates, and (5) the emergence of allosteric pockets that influence other binding sites [79]. In this study, we provide an atomic-level description of the odorant-binding mechanism to OBPs and demonstrate that it belongs to class (3) as the fluctuation of amino acids control the binding.The binding process is based on the opening of a gate formed by residues Y82 and S34. The gate remains closed thanks to a hydrogen bond between the hydroxyl group of the tyrosine and the carbonyl of the serine backbone.

We demonstrate that it is possible to design a protein that does not bind to any ligand by generating a disulfide bond through site-directed mutagenesis. The modified S34C, Y82C protein is no longer able to bind three different odorants. This confirms that the ligand accesses the binding site via a unique conserved pathway. Moreover, we also show that the binding could be controlled by distal mutations on residues whose movement correlates with this gate. A genome wide analysis show that this result could be extended to other proteins from the lipocalin family.

The remarkable stability of mammalian OBPs has been demonstrated in numerous studies, highlighting that these proteins retain their odorant-binding properties under extreme temperature conditions [32, 74, 80]. This feature is particularly crucial for using these proteins as olfactory or molecular sensors for electronic noses. Therefore, this fine description of ligand binding will open the way to rationally modulate their recognition spectrum to target specific molecules.

## Electronic supplementary material

Below is the link to the electronic supplementary material.

Supplementary Material 1

Supplementary Material 2

## Data Availability

All data are available in the main text or the supplementary materials. All MD trajectories are available upon request.
