# Supplementary material for: How allosteric mutations control ligand binding in Lipocalin protein: odorant binding protein as a test case
Source: Cell Mol Life Sci. 2025 Jun 23;82(1):250. doi: 10.1007/s00018-025-05777-8 (PMC12185845; doi:10.1007/s00018-025-05777-8)
Supplement: Supplementary file 2 — Supplementary Material 2 [file 18_2025_5777_MOESM2_ESM.docx]

How Allosteric Mutations Control Ligand Binding in Lipocalin Protein: Odorant Binding Protein as a Test Case

Maxence Lalis^1,†^, Lucie Moitrier^2,†^, Miriam Jäger^3^, Cornelia Meinert^1^, Marine Brulé^2^, Christine Belloir^2^, Nykola C. Jones^4^, Søren V. Hoffmann^4^, Sébastien Fiorucci^1^, Steffen Wolf^3^, Loïc Briand^2,^*, Jérémie Topin^1,^*

**Supplementary material**

**
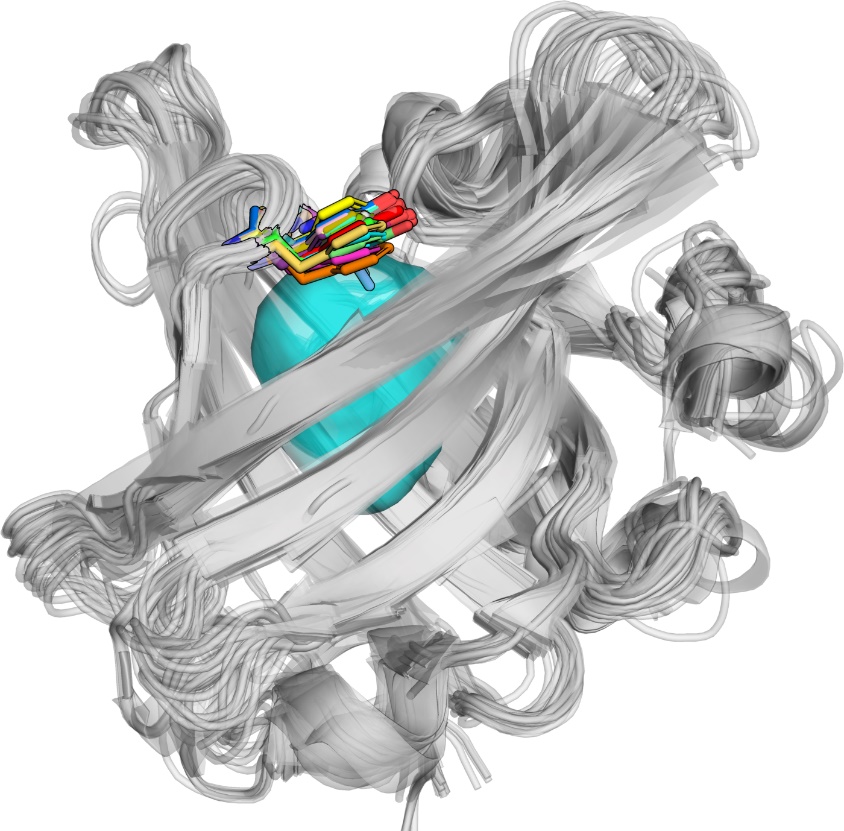
**

**Fig. S1. Conservation of the gate orientation and the calyx in experimental structures of lipocalin.** Ensemble view of the lipocalin fold highlighting the gate occluding the pocket. The protein backbone is shown in semi-transparent gray ribbons (representing 50 lipocalins structures; pdb accession codes: 1a3y, 1beb, 1bj7, 1df3, 1dzj, 1dzk, 1dzm, 1dzp, 1e00, 1e02, 1e5p, 1e06, 1ew3, 1gm6, 1i05, 1qy0, 1qy1, 1qy2, 1yp6, 1yup, 1znd, 1zne, 1zng, 1znh, 1znk, 1znl, 2a2g, 2a2u, 2akq, 2dm5, 2ozq, 2r73, 2ra6, 3fiq, 3kff, 3kfg, 3kfh, 3kfi, 3vko, 3zq3, 4wfu, 4wfv, 5gzd, 5gze, 5w1m, 5x7y, 6nre, 8a0d, 8aei, 8aej), while the binding cavity is rendered as a cyan surface. The representative conformations of the residues acting as a gate (the majority is a tyrosine) appears in different colors, illustrating the consistency of the closed-conformation across the lipocalin experimental structures.


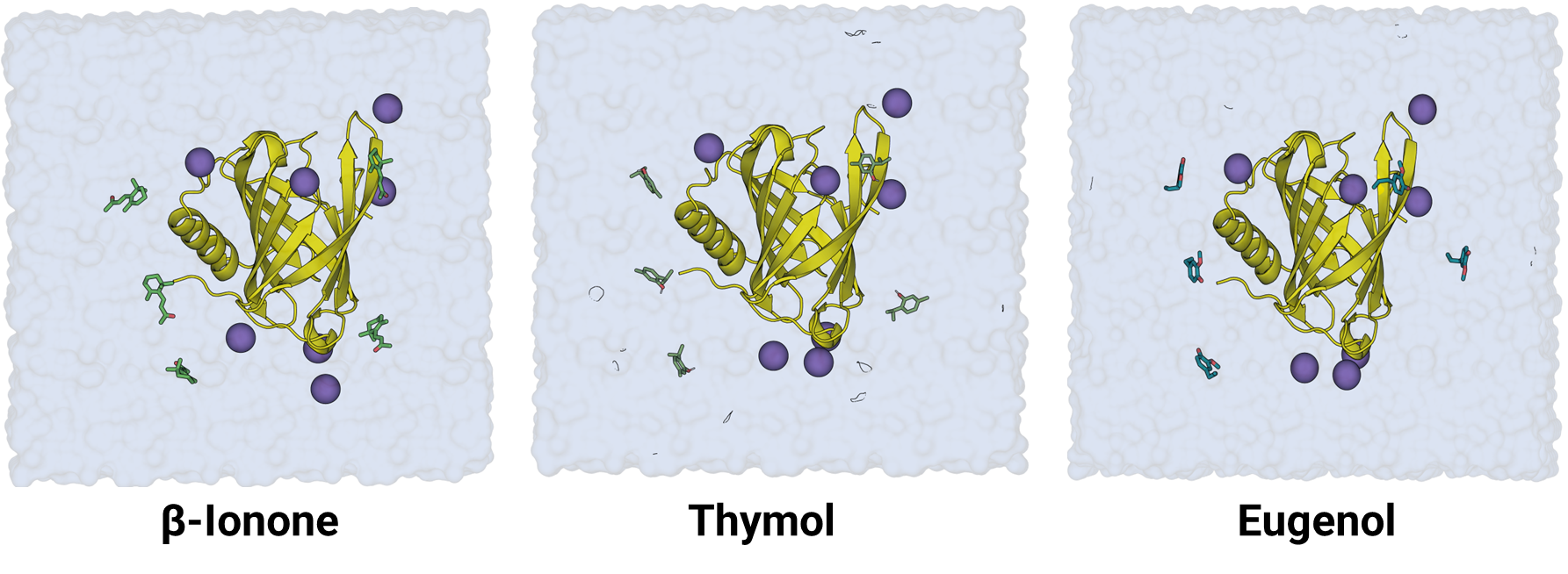


**Fig. S2. Initial coordinates of the system prior to simulation.** rOBP1 is depicted in yellow cartoon representation. Na^+^ ions appear in purple and respective ligands is in green licorice.


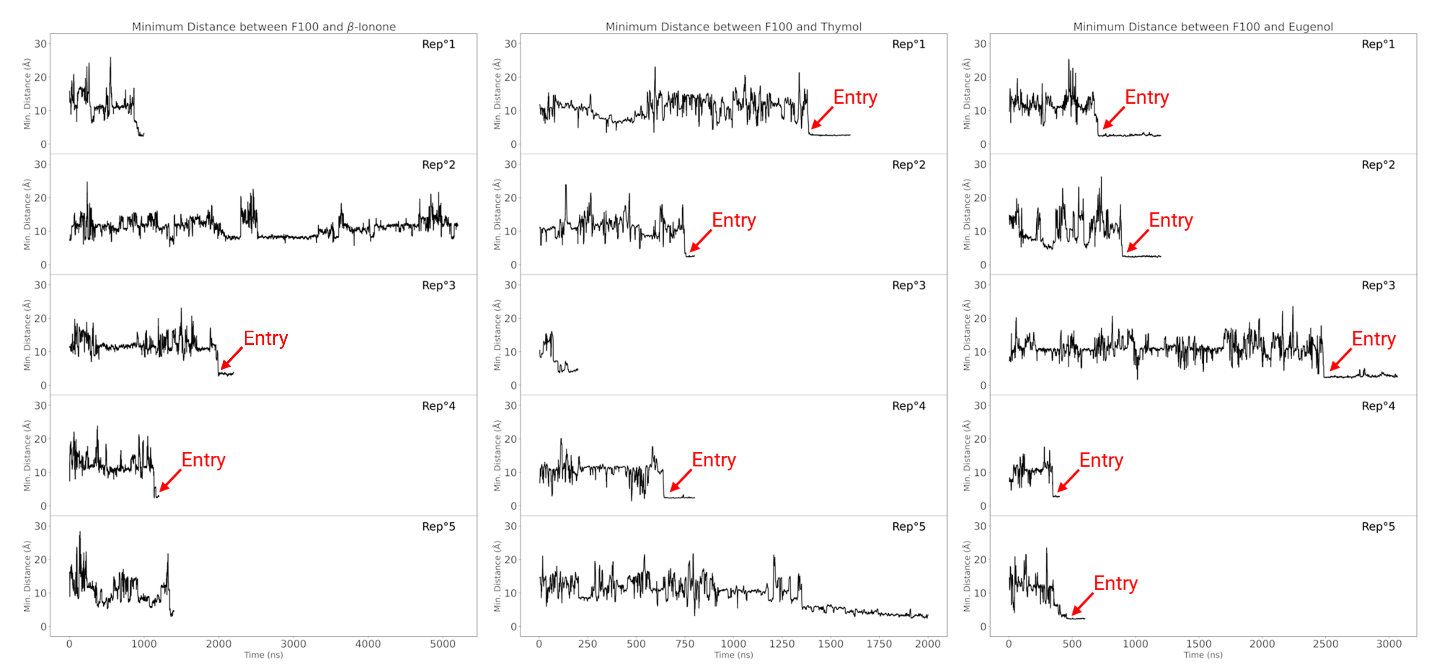


**Fig. S3. Minimum distance between the cradle of the cavity and the ligand during MD simulations of binding of the three ligands**. The minimum distance is computed between all heavy atoms from residue F100 (defined as the cradle of the cavity) and the heavy atoms of the considered ligand. The ligand is considered bound when the minimum distance is lower than 5 Å. Entries in the calyx are denoted by the red arrows.


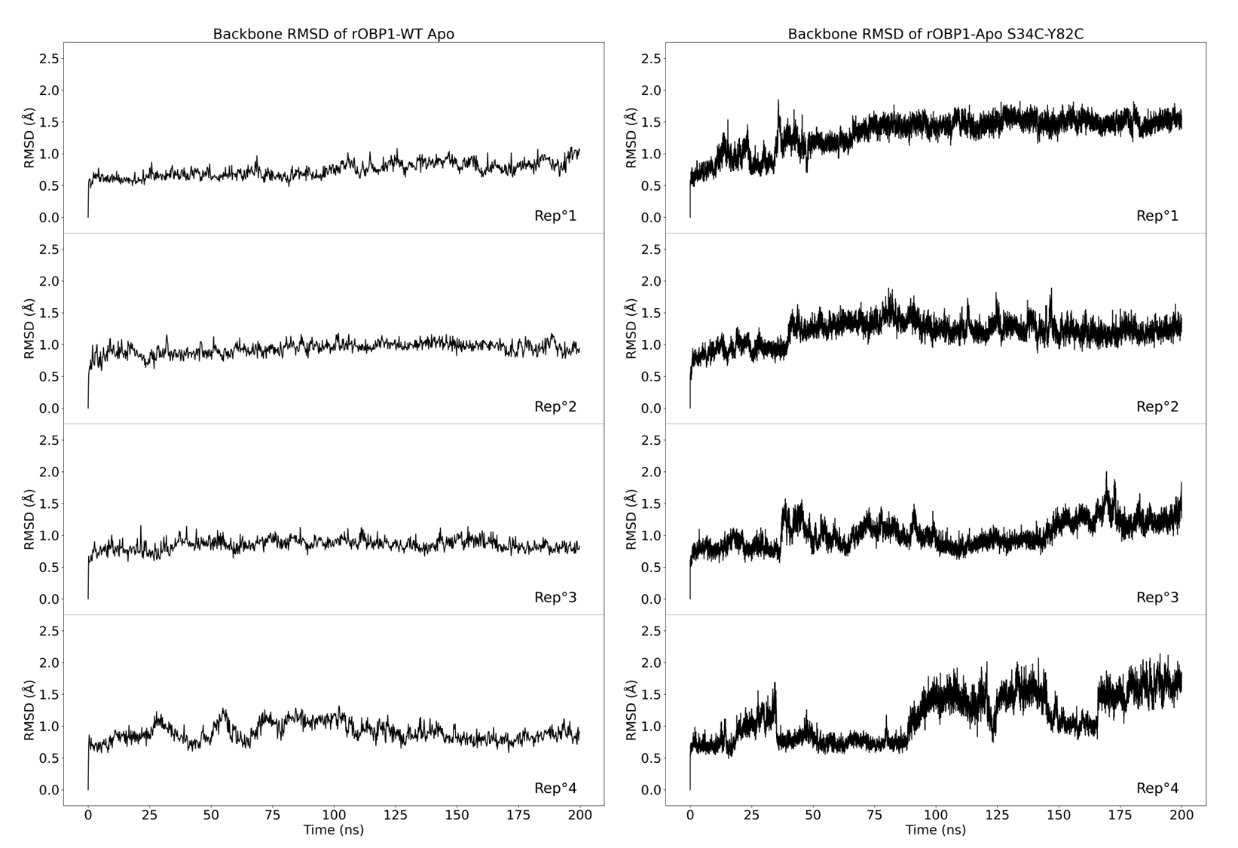


**Fig. S4. Stability of the rOBP1-wt and rOBP1 S34C-Y82C mutant in apo form in 4 replicas**. The RMSD is computed for the protein β-sheets backbone (CA, C, O, N atoms) with respect to the experimental structure for the wt (pdb code 3fiq) and initial model for the double mutant.


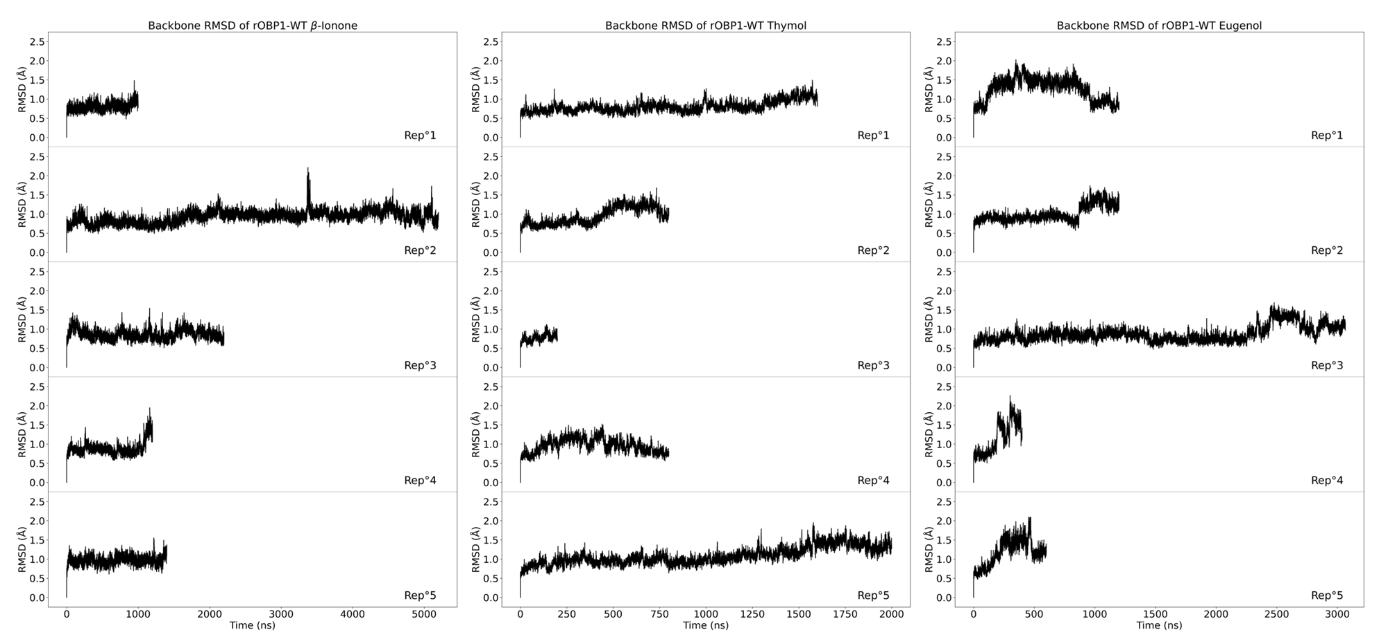


**Fig. S5.** **Stability of the rOBP1-wt protein during MD simulations with 3 different ligands (β-ionone, thymol and eugenol) in 5 replicas**. The RMSD is computed for the protein β-sheets backbone (CA, C, O, N atoms) with respect to the experimental structure (pdb code 3fiq).


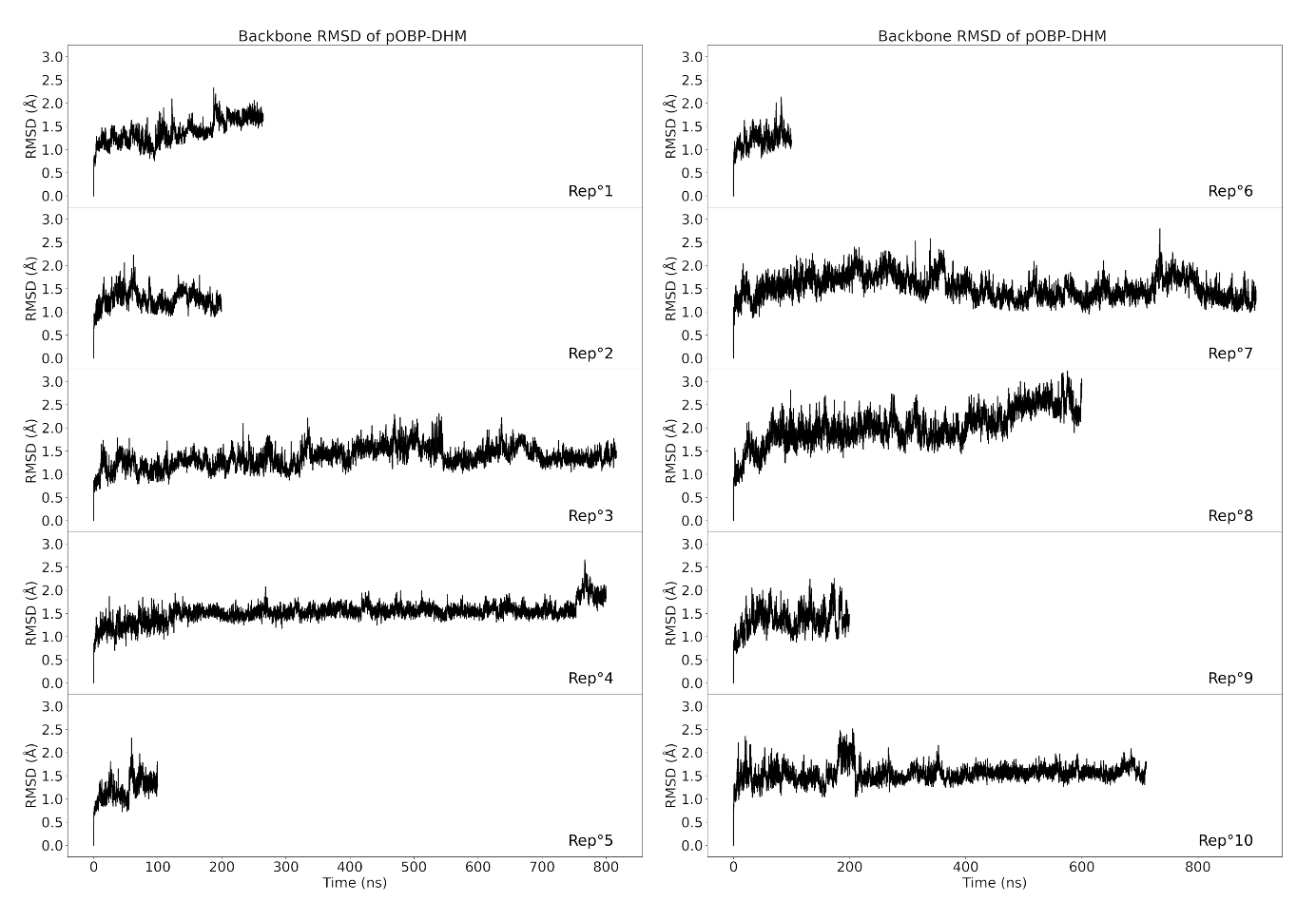


**Fig. S6.** **Stability of the pOBP1-wt protein during MD simulations with dihydromyrcenol (DHM)**. The RMSD is computed for the protein β-sheets backbone (CA, C, O, N atoms) with respect to the experimental structure (pdb code 1e00).


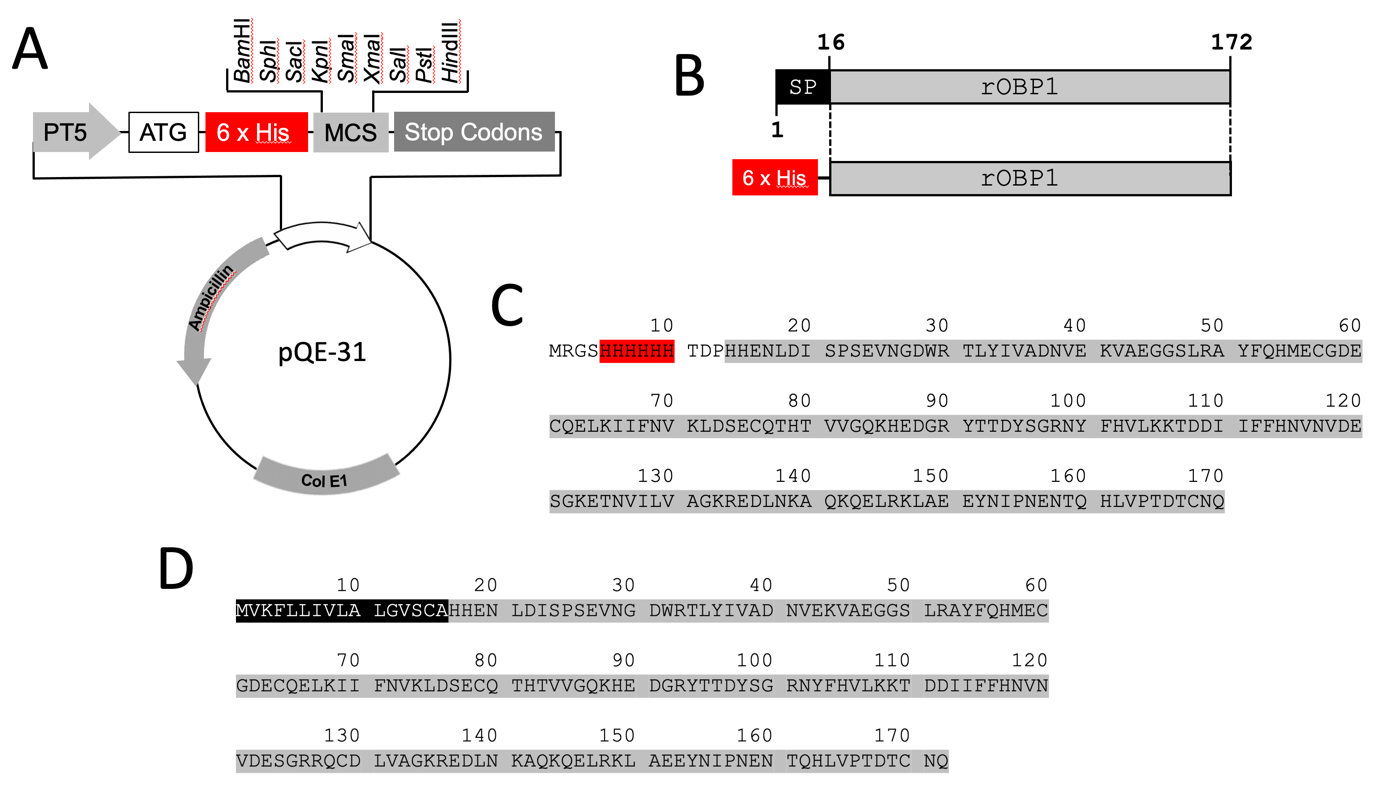


﻿**Fig. S7. Expression of the rat OBP1 in *E. coli*.** (A) The pQE-31 plasmid used for rOBP1 expression. The *E. coli* optimized cDNA sequence of rOBP1 was cloned between the *BamH*I and *Hind*III restriction sites of pQE-31. The vector contains a His6-tag sequence upstream of the mature OBP encoding sequence, PT5: T5 promoter; ATG: start codon; MCS: multicloning site, Col E1: Col E1 origin of replication, Ampicillin: ampicillin resistance gene. (B) Rat OBP1 (rOBP1) was expressed as a mature protein (UniProt ID P08937) in absence of its signal peptide (SP). The expressed protein incorporates an N-terminal 6 x His tag to help purification and detection of the protein. The numerical positions of the amino acid residues of the expressed rOBP1 are indicated. (C) Amino acid sequence of recombinantly expressed rOBP1. His6-Tag and mature amino acid sequences of rOBP1-wt (Wild-Type) are shown in red and gray, respectively. (D) Amino acid sequence of native rOBP1. The signal peptide and the mature sequence are shown in black and gray, respectively. The numerical positions of the amino acid residues of native rOBP1 are indicated.


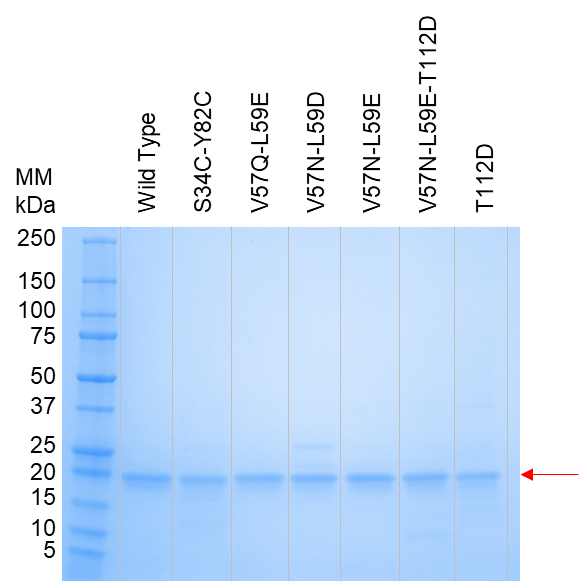


**Fig. S8. SDS-PAGE analysis of rOBP1-wt (Wild-Type) and mutants expressed in E. coli using the pQE-31 vector.** The red arrow indicates position of purified proteins.


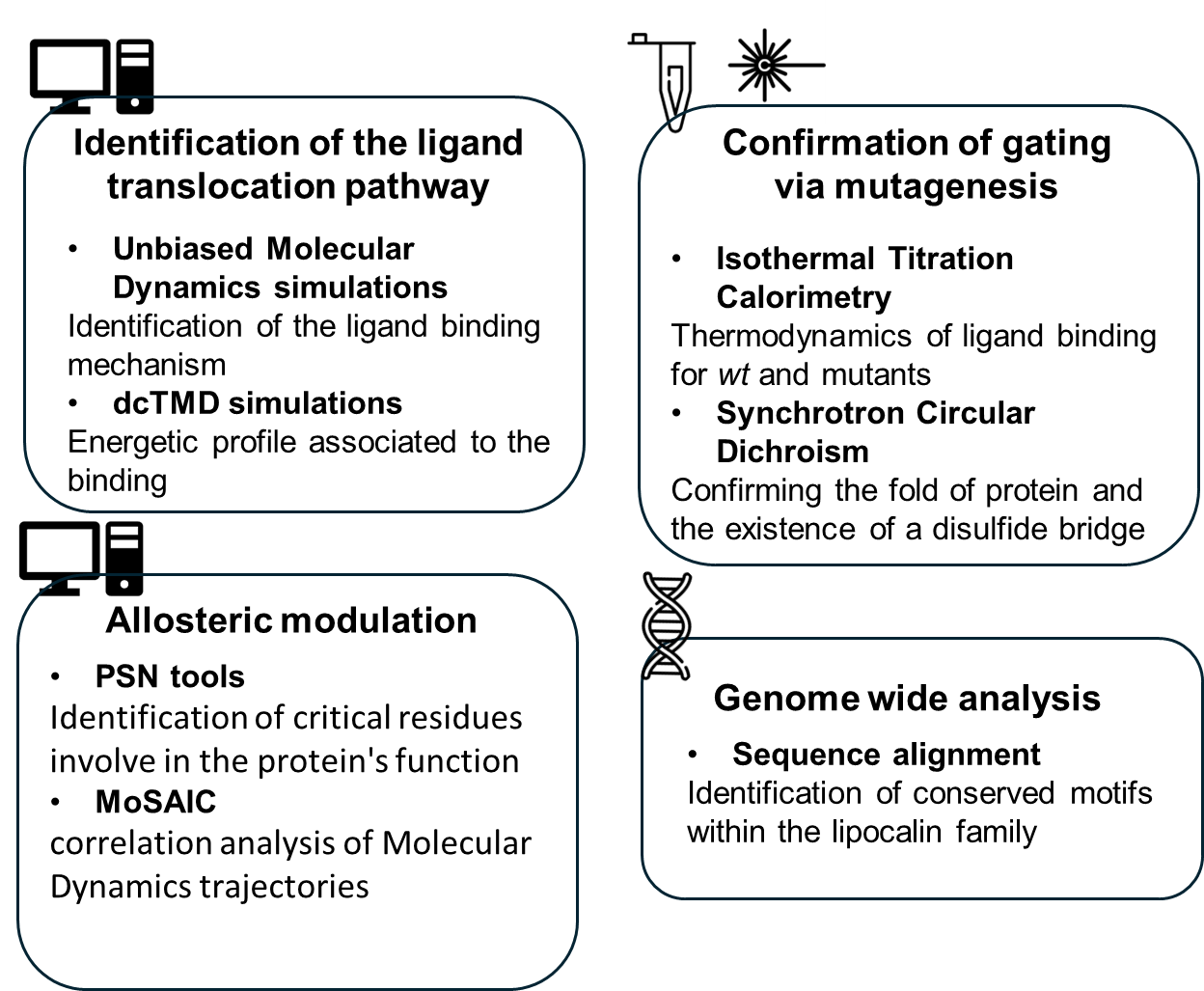


**Fig. S9. Summary of the workflow used in this study.**


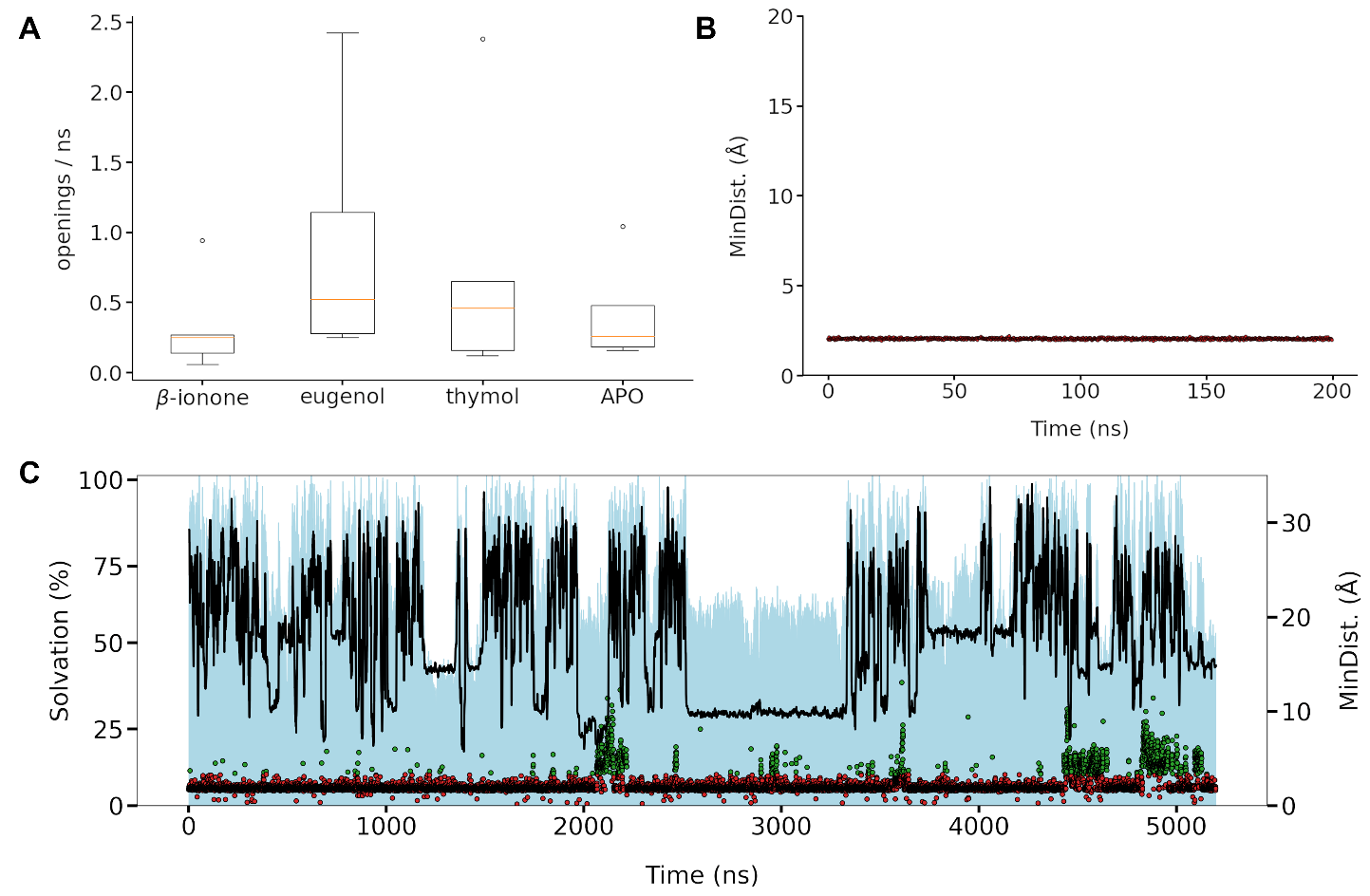


**Fig. S10. Dynamics of the gate during different simulations.** (**A**) Boxplot representation of the gate frequency openings for the different ligands (β-ionone, eugenol and thymol) and in the apo form. Frequencies, expressed as the number of openings per nanosecond, were calculated from concatenated simulations across the four conditions. Statistical analysis using the Mann-Whitney U test revealed no significant differences (p-value > 0.05). (**B**) Time evolution of gate opening during the simulation of the double mutant S34C-Y82C. Gate opening was assessed by monitoring the distance between the sulfur atoms of C34 and C82. (**C**) Evolution of gate opening and ligand solvation during a non-binding trajectory of β-ionone with rOBP1. The distance between the hydroxyl group of Y82 and the carbonyl backbone of S34 is shown as dots. Red dots indicate the closed state (<3.4 Å), while green dots represent the open state.


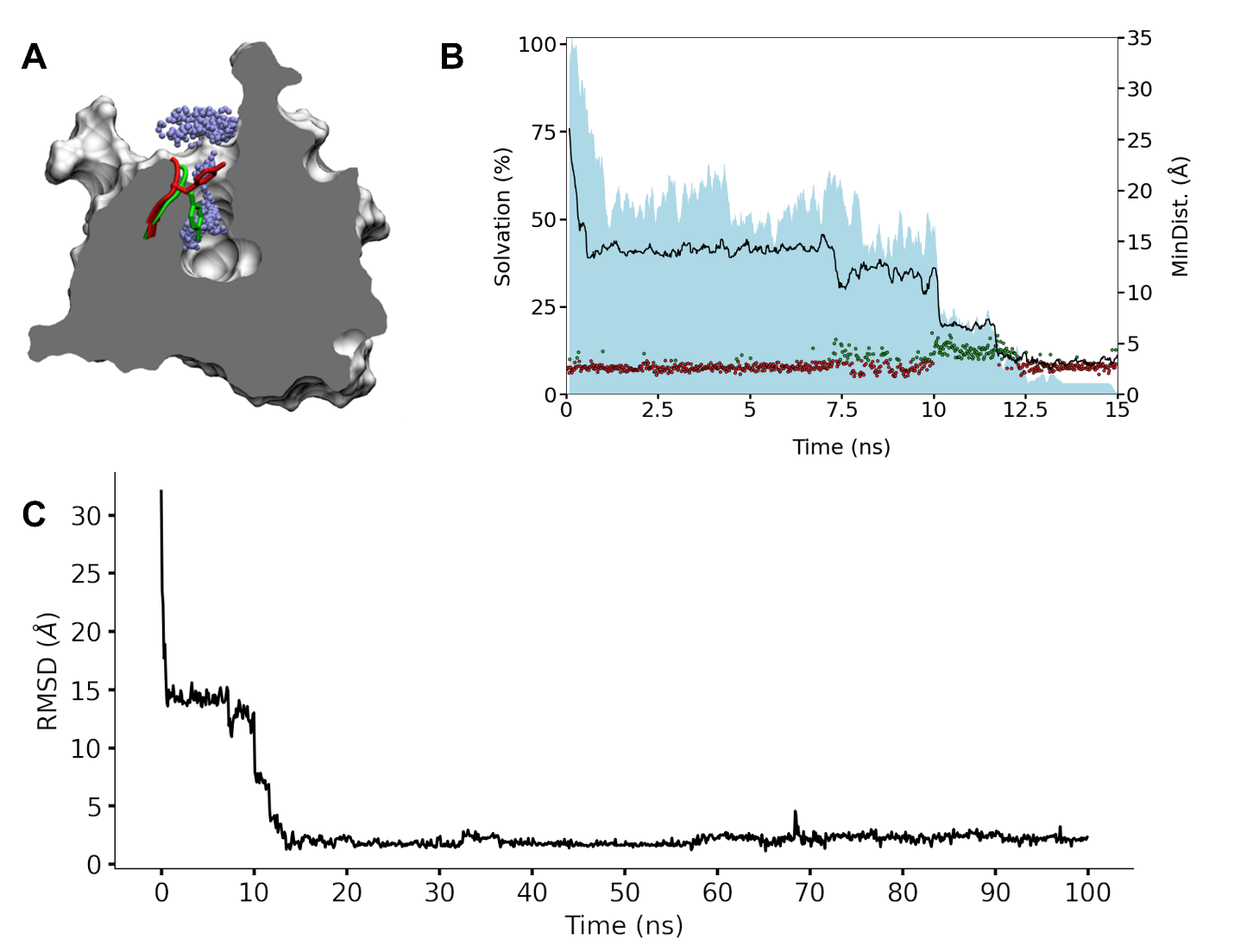


**Fig. S11. Analysis of the of the binding during MD simulation of pOBP1.** (**A**) Representation of the sliced surface of pOBP1. The calix appears in the center of the protein. The tyrosine gate is colored in red for the closed state and in green for the open state. The center of mass of dihydromyrcenol at each time step is represented by lavender spheres. (**B**) Time evolution of the distance between the centers of mass of dihydromyrcenol and the F88 side chain, represented by a black curve. The blue area represents the percentage solvation of the ligand throughout the binding process. The distance between the hydroxyl group of Y82 and the carbonyl of the P34 backbone is represented by dots, with red dots indicating the closed state (<3.4 Å) and green dots the open state. (**C**) Evolution of the RMSD of dihydromyrcenol during the simulation. The RMSD was calculated on the heavy atoms of dihydromyrcenol, using the experimental structure (pdb code: 1e00) as reference.


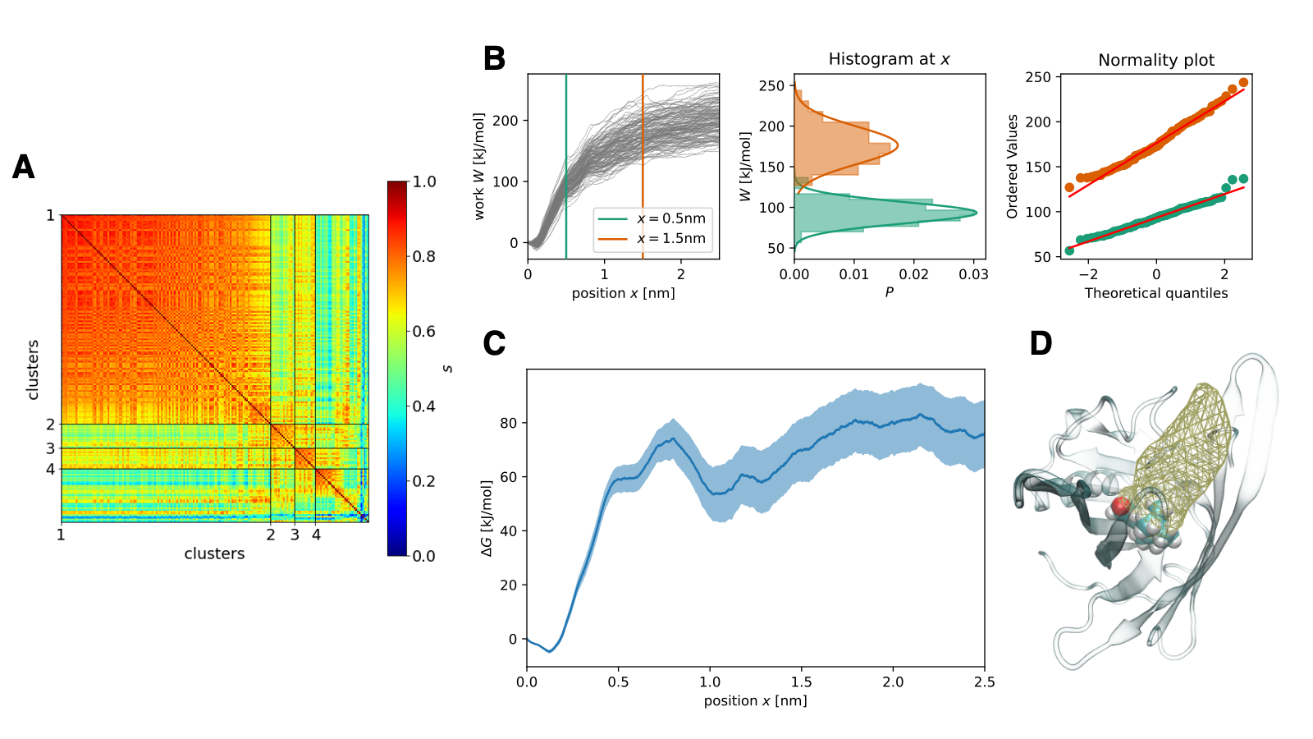


**Fig. S12. Trajectory clustering and pathway analysis**. (**A**) Trajectory clustering according to highest similarity. The largest cluster, numbered 1, contains 130 of the 200 simulated unbinding events. (**B**) Normality analysis of cluster 1 trajectories. The work distribution is reasonably close to a normal distribution, allowing for dissipation correction. (**C**) Error estimate of free energy profile. The shaded area represents the bootstrap error estimate from 1000 bootstrap draws. (**D**) Ligand occupancy density for cluster 1 during unbinding simulations. The biased unbinding pathway coincides with the binding pathway in unbiased simulations.

**
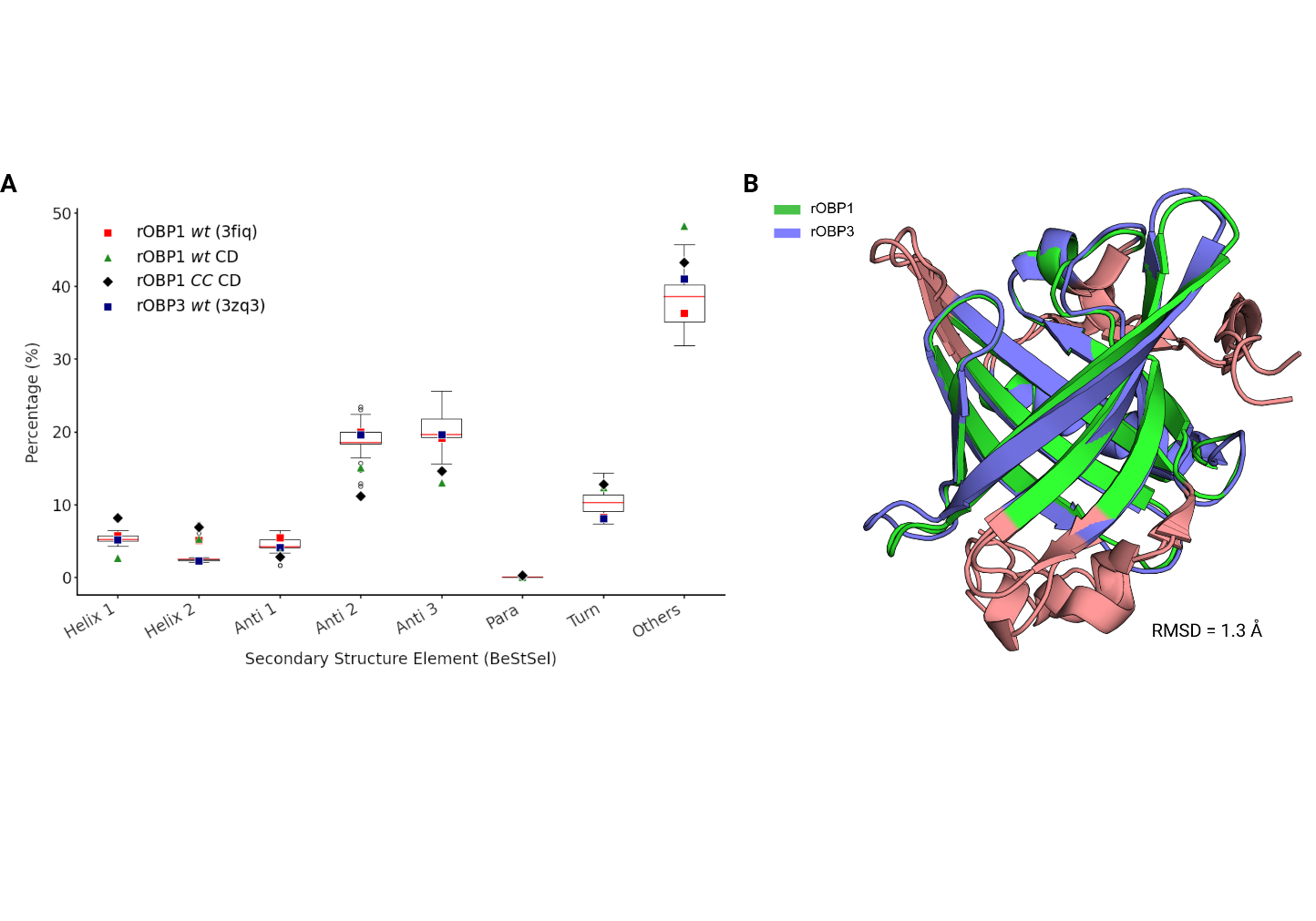
**

**Fig. S13. Structure analysis of different OBP.** (**A**) Boxplot representation of the percentage of secondary structures for different lipocalins extracted from the experimental structures (50 lipocalins structures; pdb accession codes: 1a3y, 1beb, 1bj7, 1df3, 1dzj, 1dzk, 1dzm, 1dzp, 1e00, 1e02, 1e5p, 1e06, 1ew3, 1gm6, 1i05, 1qy0, 1qy1, 1qy2, 1yp6, 1yup, 1znd, 1zne, 1zng, 1znh, 1znk, 1znl, 2a2g, 2a2u, 2akq, 2dm5, 2ozq, 2r73, 2ra6, 3kff, 3kfg, 3kfh, 3kfi, 3vko, 4wfu, 4wfv, 5gzd, 5gze, 5w1m, 5x7y, 6nre, 8a0d, 8aei, 8aej) using BeStSell. The values from the spectral deconvolution appears as a black diamond, a blue square and a green triangle for rOBP1, rOBP3 and rOBP1 CC respectively. (**B**) Comparison of the two experimental structures of rOBP1 (green) and rOBP3 (blue). The two structures are highly similar with a rmsd calculated on the backbone of 1.3 Å. Regions with the highest deviations are highlighted in pink.


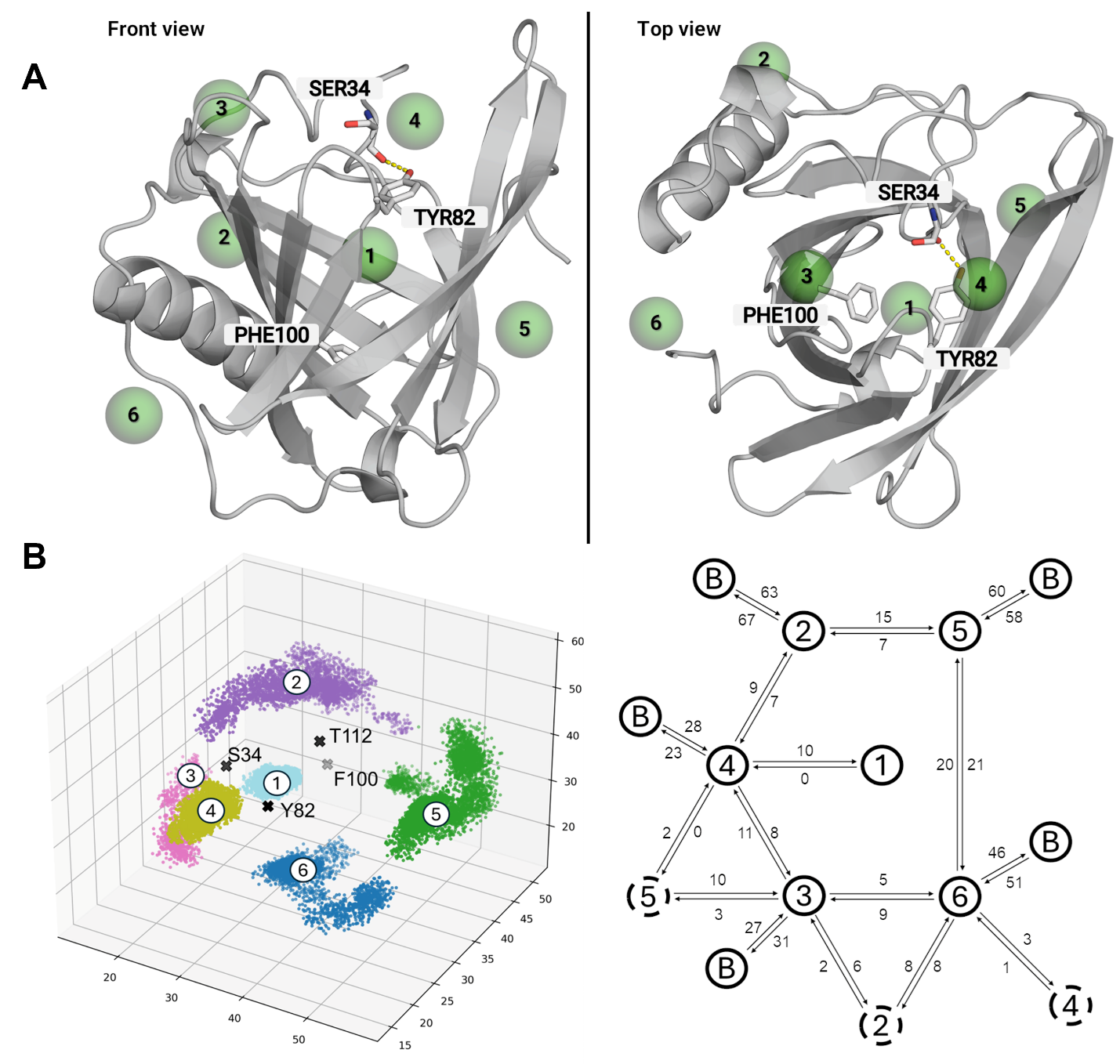


**Fig. S14.** **Aggregate positions of ligands during MD simulations**. All simulations have been concatenated before cluster analysis. (**A**) Ligand occupancy clusters mapped onto the structure of rOBP1. Each cluster is represented by a green sphere numerated from 1 to 6. The gate comprised of residue S34 and Y82 as well as the cradle of the cavity, F100, are represented in licorice. (**B**) Left, 3D mapping of the different ligands occupancy clusters. Cluster 1 represents the sampling of the calyx and cluster 3 and 4 are the two areas that were subjected to mutagenesis analysis. On the right is displayed the number of transitions between each cluster. A ligand was considered to be in a specific cluster if it remains in it during more than 100 ps. The letter B accounts for the bulk.


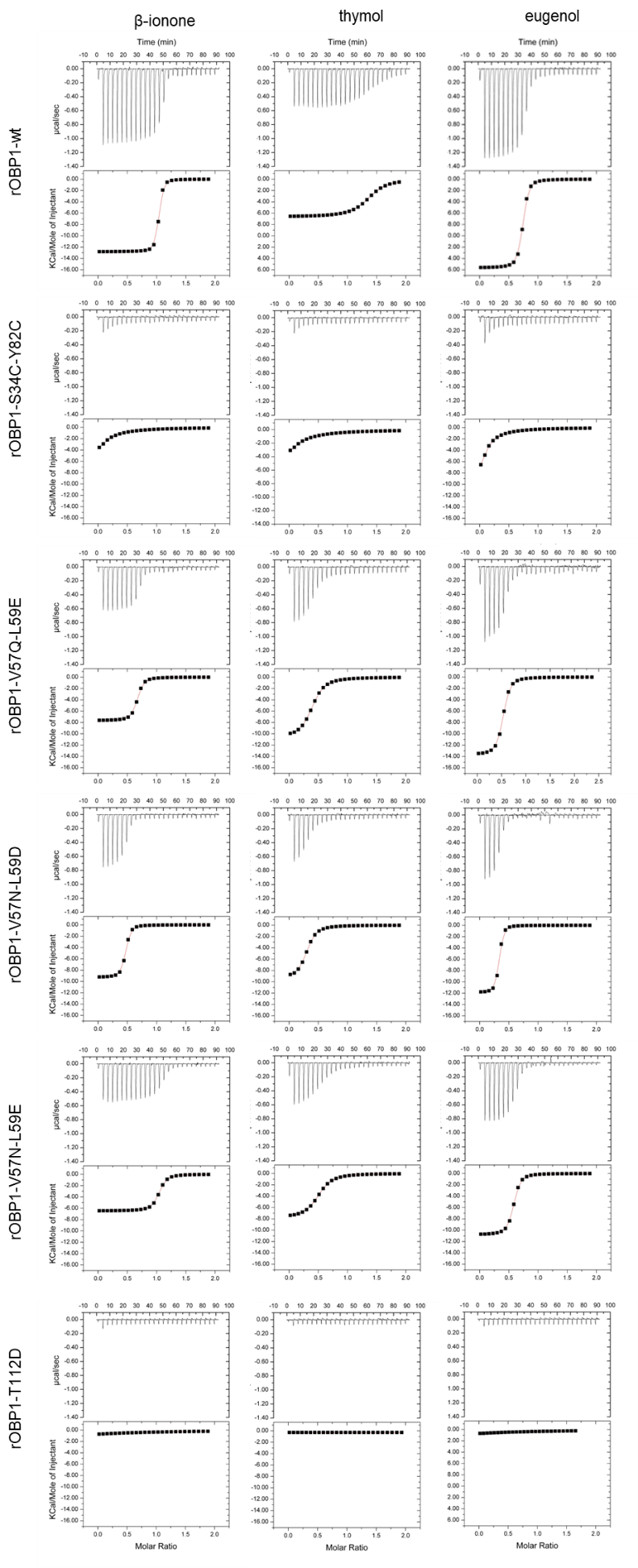


**Fig. S15. Binding properties of rOBP1 wt and mutant to the three considered odorants (β-ionone, thymol and eugenol) evaluated by isothermal titration calorimetry.** The top panels are the thermograms, and the bottom panels are the fitted binding isotherms.

**Correlation Analysis**

*Choice of Contacts*

In this study, contact formation is assumed when the distance *d_ij_* between the nearest non-hydrogen atoms of residues *i* and *j* falls below 4.5 Å. To mitigate potential noise, only resid 4 to 120 are used and the three neighboring residues within the chain are excluded. Additionally, a prerequisite for contact inclusion is a minimum population threshold of 5% across all trajectories, resulting in the identification of a total of 408 contacts.

*Correlation analysis*

First, the minimal distances between contacts is computed, excluding hydrogen atoms. Next, the Pearson

correlation coefficients *ρ* are calculated between the minimal distances via:

$$\rho\left( X,Y \right)=\frac{\left\langle\left( X-\left\langle X \right\rangle\right)\left( Y-\left\langle Y \right\rangle\right) \right\rangle}{\sigma_{X}\sigma_{Y}}$$

where X,Y are distance vectors, $\left\langle\ldots\right\rangle$ denotes the statistical average and *σ* the variance. *ρ* can have values between -1 and 1. With -1 meaning anticorrelated, 0 no linear correlation and 1 completely correlated. *ρ* is determined for all the contacts using the mosaic tool(**Diez et al, 2022**). At last, the |*ρ_ij_*| are clustered using the Leiden community detection algorithm (**Diez et al, 2022)** with the constant potts model (CPM)

$$\Phi=\sum_{c} \left[ e_{c}-\gamma\left( \begin{matrix} n_{c} \\ 2 \end{matrix} \right) \right]$$

As objective function. Where *n_c_* denotes the number of contacts in cluster *c* and $\left( \begin{matrix} n_{c} \\ 2 \end{matrix} \right)$ the number of edges. The resolution parameter *γ* weights this number. Thus, Eq. II.2 compares the total correlation within a cluster *e_c_* with a hypothetical cluster of same size with the *γ* correlation .


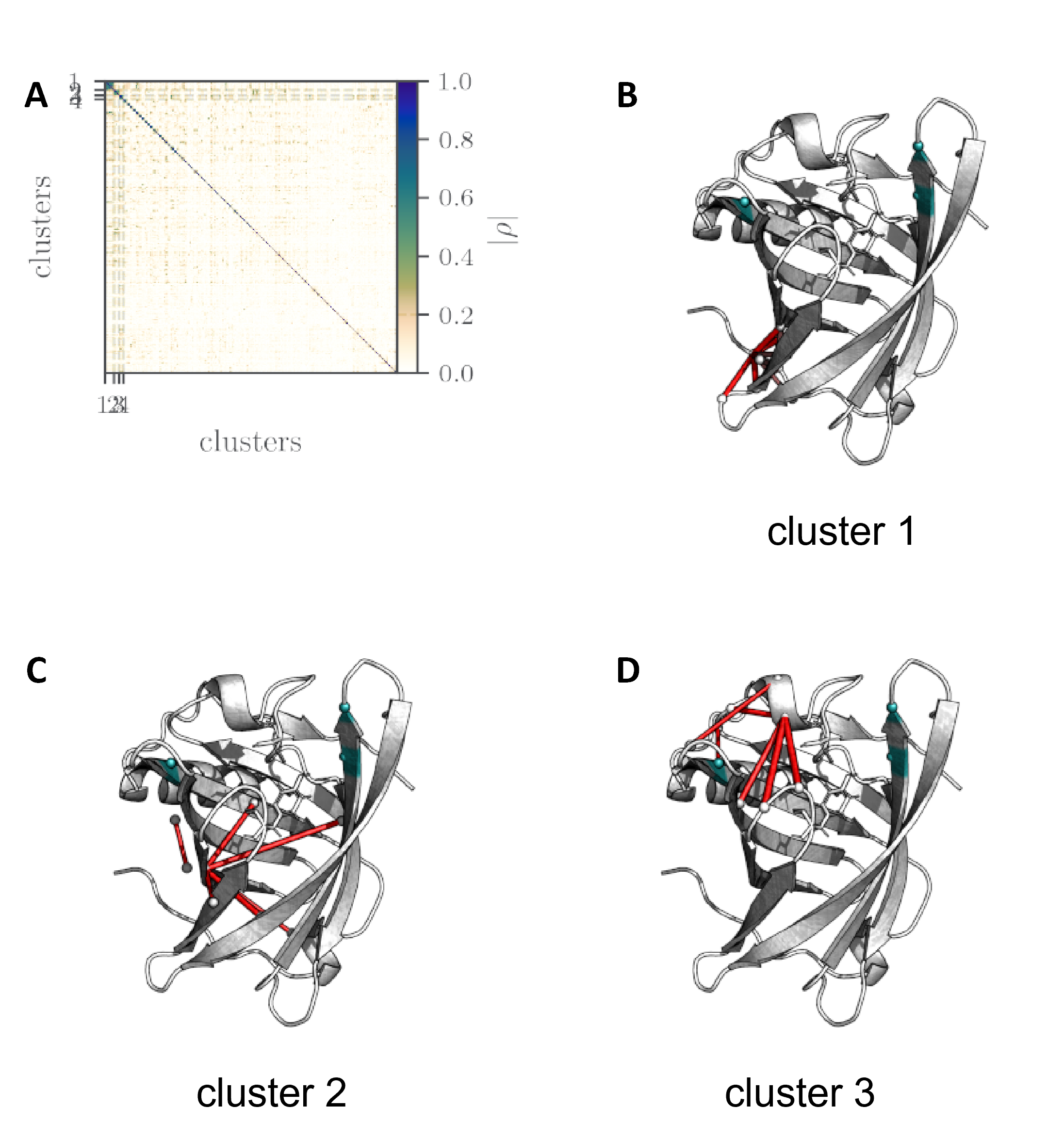


**Fig. S16. Mosaic analysis of correlated dynamics.** (**A**) Block matrix after community analysis with a resolution parameter $\gamma$= 0.2. Only three small correlated communities (clusters 1 to 3) of correlated dynamics can be observed, while most minimal contact distances are uncorrelated (cluster 4). (**B-D**) Positions of the five strongest correlations within clusters 1 to 3 highlighted as red sticks within a cartoon representation of OBP. Stick thickness indicates the strength of correlation.

**Table S1. Previously mutated residues from different mammalian OBP.**

| OBP | Mutation | Reference |
| --- | --- | --- |
| pOBP1 | W16F, F88W, I42L, I100L, F35A, Y82A | **[Nagnan-Le Meillour et al., 2014](#_ENREF_96)**, **Mastrogiacomo et al., 2014b**, **Wei et al., 2008**, **Zaremska et al., 2020** |
| hOBPIIa | K62A, K82A, K112A | [**Tcatchoff et al., 2006**](#_ENREF_95) |
| aimelOBP3 | N90L, E120A, S122A | **Zhu et al, 2017** |

**Table S2.** **Overview of ligand-receptor interaction frequency for eugenol, thymol and β-ionone during the** **rOBP1 simulations**. Hydrophobic accounts for hydrophobic interaction between the ligand and an amino acid while HBAcceptor accounts for the hydrogen bond formed between an amino acid and the ligand. Frequency is calculated by considering frames of simulation when the ligand is inside the calyx (distance < 5 Å between F100 and the ligand). Positions highlighted in bold were identified as interacting with ligands in previous studies.

|  |  | **Frequency (%)** | | |
| --- | --- | --- | --- | --- |
| **Position** | **Interaction** | β-ionone | Thymol | Eugenol |
| I21 | Hydrophobic | 47 | 23 | 7 |
| **L35** | Hydrophobic | 85 | 62 | 54 |
| A37 | Hydrophobic | 69 | 72 | 74 |
| F39 | Hydrophobic | 82 | 83 | 83 |
|  | PiStacking | 0 | 40 | 54 |
| I53 | Hydrophobic | 83 | 63 | 43 |
| F55 | Hydrophobic | 83 | 66 | 80 |
| V68 | Hydrophobic | 85 | 53 | 71 |
| T80 | Hydrophobic | 50 | 18 | 20 |
| **Y82** | Hydrophobic | 81 | 35 | 56 |
| **N86** | Hydrophobic | 41 | 29 | 26 |
| **F88** | Hydrophobic | 91 | 87 | 64 |
| **F100** | Hydrophobic | 46 | 68 | 42 |
| N102 | Hydrophobic | 72 | 87 | 82 |
|  | HBAcceptor | 97 | 13 | 3 |
| V114 | Hydrophobic | 73 | 76 | 37 |
| **L116** | Hydrophobic | 86 | 94 | 75 |

**Table S3. Isothermal titration calorimetry (ITC) data for wild-type (WT) and mutant rOBP1 binding different odorants.** Each row corresponds to a different mutant and odorant combination. The columns list the binding stoichiometry (N), the dissociation constant (k_D_), the enthalpy change (ΔH), the entropy change (ΔS), and the Gibbs free energy (ΔG). The last columns compare these values to the wild-type reference (Δk_D_, ΔΔH, and ΔΔS). Red or blue shading highlights positive or negative differences relative to wild-type.

| **rOBP1** | **Odorants** | **N (sites)** | **k_D_ (µM)** | **ΔH (cal/mole)** | **ΔS (cal/mole/deg)** | **ΔG** | **Δk_D_** | **ΔΔH (cal/mole)** | **ΔΔS (cal/mole/deg)** |
| --- | --- | --- | --- | --- | --- | --- | --- | --- | --- |
| WT | β-ionone | 1.0 | 0.019 | -1.28E+04 | -7.7 | -1.26E+04 | - | - | - |
|  | Thymol | 1.4 | 0.441 | -6.64E+03 | 6.8 | -6.81E+03 | - | - | - |
|  | Eugenol | 0.7 | 0.060 | -1.57E+04 | -19.4 | -1.52E+04 | - | - | - |
| V57Q-L59E | β-ionone | 0.6 | 0.090 | -7.67E+03 | 6.5 | -7.83E+03 | 0.071 | 5.14E+03 | 14.17 |
|  | Thymol | 0.4 | 0.775 | -1.08E+04 | -8.2 | -1.06E+04 | 0.335 | -4.13E+03 | -14.98 |
|  | Eugenol | 0.5 | 0.164 | -1.37E+04 | -14.9 | -1.33E+04 | 0.105 | 1.94E+03 | 4.5 |
| V57N-L59D | β-ionone | 0.4 | 0.076 | -9.27E+03 | 1.5 | -9.30E+03 | 0.056 | 3.54E+03 | 9.15 |
|  | Thymol | 0.3 | 0.599 | -9.49E+03 | -3.3 | -9.40E+03 | 0.158 | -2.85E+03 | -10.15 |
|  | Eugenol | 0.3 | 0.069 | -1.19E+04 | -7.1 | -1.17E+04 | 0.010 | 3.76E+03 | 12.27 |
| V57N-L59E | β-ionone | 1.0 | 0.089 | -6.47E+03 | 10.6 | -6.73E+03 | 0.070 | 6.34E+03 | 18.26 |
|  | Thymol | 0.5 | 0.746 | -7.84E+03 | -1.7 | -7.80E+03 | 0.306 | -1.20E+03 | -8.53 |
|  | Eugenol | 0.6 | 0.123 | -1.08E+04 | -4.6 | -1.07E+04 | 0.063 | 4.84E+03 | 14.78 |
